# c-Met Signaling Protects from Nonalcoholic Steatohepatitis- (NASH-) Induced Fibrosis in Different Liver Cell Types

**DOI:** 10.1155/2018/6957497

**Published:** 2018-11-12

**Authors:** Hannah K. Drescher, Fabienne Schumacher, Teresa Schenker, Maike Baues, Twan Lammers, Thomas Hieronymus, Christian Trautwein, Konrad L. Streetz, Daniela C. Kroy

**Affiliations:** ^1^Department of Internal Medicine III, University Hospital, RWTH, Aachen, Germany; ^2^Institute for Experimental Molecular Imaging, University Hospital, RWTH, Aachen, Germany; ^3^Institute for Biomedical Engineering-Cell Biology, University Hospital, RWTH, Aachen, Germany

## Abstract

Nonalcoholic steatohepatitis (NASH) is the most common chronic, progressive liver disease in Western countries. The significance of cellular interactions of the HGF/c-Met axis in different liver cell subtypes and its relation to the oxidative stress response remains unclear so far. Hence, the present study is aimed at investigating the role of c-Met and the interaction with the oxidative stress response during NASH development in mice and humans. Conditional c-Met knockout (KO) lines (LysCre for Kupffer cells/macrophages, GFAPCre for *α*-SMA^+^ and CK19^+^ cells and MxCre for bone marrow-derived immune cells) were fed chow and either methionine-choline-deficient diet (MCD) for 4 weeks or high-fat diet (HFD) for 24 weeks. Mice lacking c-Met either in Kupffer cells, *α*-SMA^+^ and CK19^+^ cells, or bone marrow-derived immune cells displayed earlier and faster progressing steatohepatitis during dietary treatments. Severe fatty liver degeneration and histomorphological changes were accompanied by an increased infiltration of immune cells and a significant upregulation of inflammatory cytokine expression reflecting an earlier initiation of steatohepatitis development. In addition, animals with a cell-type-specific deletion of c-Met exhibited a strong generation of reactive oxygen species (ROS) by dihydroethidium (hydroethidine) (DHE) staining showing a significant increase in the oxidative stress response especially in LysCre/c-Met^mut^ and MxCre/c-Met^mut^ animals. All these changes finally lead to earlier and stronger fibrosis progression with strong accumulation of collagen within liver tissue of mice deficient for c-Met in different liver cell types. The HGF/c-Met signaling pathway prevents from steatosis development and has a protective function in the progression to steatohepatitis and fibrosis. It conveys an antifibrotic role independent on which cell type c-Met is missing (Kupffer cells/macrophages, *α*-SMA^+^ and CK19^+^ cells, or bone marrow-derived immune cells). These results highlight a global protective capacity of c-Met in NASH development and progression.

## 1. Introduction

Nonalcoholic fatty liver disease (NAFLD) is a chronic and progressive liver disease which is evolving to be one of the leading causes for liver transplantation [1–3]. As the most common chronic liver disease in Western countries, NAFLD is accompanied by a specific sequence of hepatic alterations that begins with simple steatosis which can easily progress to more advanced stages like nonalcoholic steatohepatitis (NASH), liver fibrosis, and cirrhosis and finally the development of hepatocellular carcinoma (HCC) leading to an increased mortality [4, 5]. When considering that more than 20% of the general population in industrialized nations have a fatty liver with the risk to progress to NASH [6], the dramatic need for the development of therapeutic treatment options gets evident. A hallmark of NASH is its correlation to the metabolic syndrome and its close relationship to type II diabetes mellitus which is predominantly characterized by fat accumulation, insulin resistance, and dyslipidemia [7]. Also, on the molecular level, NASH is associated with a consecutive impairment of the metabolism and an intense inflammatory immune response. Alterations in the lipid metabolism lead to an increased secretion of free fatty acids from skeletal muscle and adipose tissue which in turn leads to the accumulation of triglycerides and other lipids in hepatocytes [8, 9]. Toxic lipids can provoke intrahepatic cell death inducing the oxidative stress and proinflammatory immune response. Histologically, the cause of NASH disease development can be observed by ballooning of hepatocytes, infiltration of immune cells, and finally the accumulation of collagen fibers within liver tissue. The progression to fibrosis can in the end provide a loss of organ function.

Met, the cellular receptor tyrosine kinase for the hepatocyte growth factor (HGF), plays a pivotal role in mediating processes like stem cell growth, wound healing, motility, and morphogenesis [10]. Previous studies could further show its importance in the growth of hepatocytes, fibrogenesis, and immune modulation [11, 12]. On the molecular level, the direct binding of HGF to its receptor c-Met leads to the homodimerization followed by phosphorylation of this receptor at the tyrosine residues Y1234 and Y1235 within the catalytic domain of the tyrosine kinase domain [13]. Phosphorylation leads to the activation of several downstream signaling pathways conveying multiple intracellular functions including the PI3K/AKT [14], JAK/STAT [15, 16], and Ras/MAPK cascades [17–19] controlling, e.g., proliferation and apoptosis [20].

The importance of c-Met gets more obvious when considering that mice with a deletion of this receptor exhibit serious developmental defects of several muscle groups [21], placenta, and liver between weeks 13 and 16 of embryogenesis up to death of these animals in utero [22–25].

c-Met is predominantly expressed on cells with endothelial and epithelial origins and has initially been described as a protooncogene [10]. In the recent review summarizing the role of c-Met as a therapeutic option in HCC, it was shown that only the selective inhibition of c-Met shows antitumoral potential in HCC, whereas the nonselective kinase inhibition with c-Met activity failed in clinical trials [26].

During chronic liver injury, especially in NASH, c-Met has been shown to play a benefitial, hepatoprotective role by suppressing chronic inflammation and progression to fibrosis [27]. The generation of conditional c-Met knockout mice first described by Borowiak et al. [28] highlighted the role of this receptor in liver regeneration. Mice with a MxCre-specific deletion for c-Met displayed reduced liver regeneration based on a cell cycle defect together with reduced ERK activity while Akt phosphorylation still occurred.

Based on the current knowledge, the aim of this study was to elucidate the particular role of c-Met on the different liver cell types during the development and progression of diet-induced steatohepatitis in two distinct murine feeding models. Mice with a deficiency of c-Met in either Kupffer cells/myeloid cells, cells under the glial fibrillary acid protein promotor, or bone marrow-derived immune cells were treated with high-fat diet (HFD) or methionine-choline-deficient diet (MCD). This study is the first to publish that the deletion of c-Met, insignificant on which liver cell types (Kupffer cells/macrophages, *α*-SMA^+^/CK19^+^ cells, or bone marrow-derived immune cells), accelerated the disease outcome in diet-induced steatohepatitis and fibrosis.

## 2. Material and Methods

### 2.1. Animal Studies

The study was carried out in accordance to the law of the regional authorities for nature, environment, and consumer protection of North Rhine-Westfalia (Landesamt für Natur, Umwelt und Verbraucherschutz NRW (LANUV), Recklinghausen, Germany) and approved by the LANUV Committee (permit number: TV11018G). All experiments were performed in accordance with the German guidelines for animal housing and husbandry.

### 2.2. Human Samples

52 liver specimens were obtained according to the local ethics committee rules from liver explantation or liver resection recruited at the RWTH Aachen University Hospital (local IRB permit number EK 166-12). When intrahepatic lesions were present, the tissue for this study was collected from the most distant section of the specimen. Overall, we analyzed 12 samples from patients with HBV, 17 HCV, 6 PBC, 8 PSC, 5 alcoholic cirrhosis, and 4 NASH patient samples.

### 2.3. Housing and Generation of Mice

Male C57BL/6J wild-type mice, conditional c-Met^fl/fl^ and LysCre c-Met^mut^, conditional c-Met^fl/fl^ and GFAPCre c-Met^mut^, and conditional c-Met^fl/fl^ and MxCre c-Met^mut^ mice all in C57BL/6J background were used as experimental animals. At least five animals per group and time point were treated in parallel. Respective c-Met^fl/fl^ animals were always littermates to the corresponding c-Met^mut^ mice. All experiments were repeated at least twice. Experimental animals were housed in the animal facility of the RWTH Aachen University Hospital with 12-hour light/dark cycles and water and food *ad libitum* available. Treatments were in accordance with the criteria of the German administrative panel on laboratory animal care.

### 2.4. Dietary Treatments

All dietary treatments were performed with 8–12-week-old male mice weighing at least 25 g. Mice were fed either with chow diet, methionine-choline-deficient diet (MCD) (Sniff, Soest, Germany), or high-fat diet (HFD) (40 kcal% fat (vegetable fats), 20 kcal% fructose, and 2% cholesterol) (Brogaarden, Lynge, Denmark). Wild-type and knockout mice showed a food intake of about 6 g per day without any differences between chow- and diet-treated animals.

Further material and methods' data are provided in Supplementary Materials (available here).

## 3. Results

### 3.1. Effective c-Met Deletion in the Different Cre Lines

To determine the specificity of the c-Met deletion in the different used Cre lines (LysCre, GFAPCre, and MxCre), we first conducted genomic PCR genotyping, detecting the recombinant and nonrecombinant c-Met allele. Analysis was performed for hepatocytes, Kupffer cells/macrophages, *α*-SMA^+^/CK19^+^ cells, and bone marrow-derived immune cells in all three tissue-specific genetic settings. These controls show the presence of the c-Met WT allele in the varying liver cell types not targeted by the specific Cre promotor (Supplementary Figure 1A). The next experiment was supposed to show the efficient deletion of c-Met in Kupffer cells/macrophages under the LysCre promotor (Supplementary Figure 1B), on *α*-SMA^+^/CK19^+^ cells under the GFAPCre promotor (Supplementary Figure 1C), and on bone marrow-derived immune cells under the MxCre promotor (Supplementary Figure 1D) at baseline conditions. All results show a deletion of c-Met on the specific cell compartment.

### 3.2. The Loss of c-Met in Kupffer Cells/Myeloid Cells Increases Steatosis Development and Inflammation in the MCD and HFD Model of Steatohepatitis

**Figure 1:**
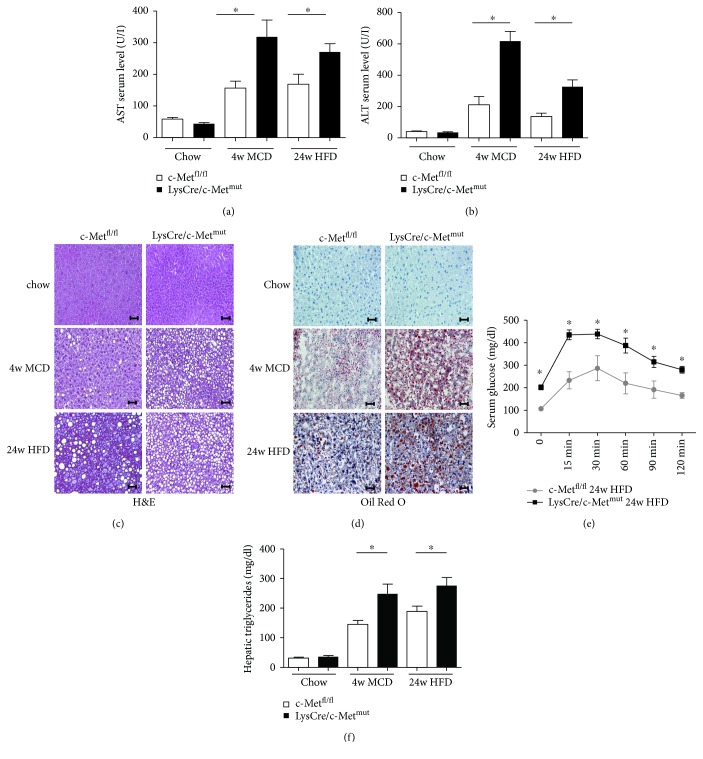
Increased steatosis in LysCre/c-Met^mut^ mice after MCD and HFD feeding (a) AST levels in serum of c-Met^fl/fl^ and LysCre/c-Met^mut^ mice after chow, MCD (4 weeks), and HFD (24 weeks) feeding. Serum transaminases increase after treatment with steatosis-induced diets (*n* = 8) (^∗^ *p* < 0.05). (b) ALT levels in serum of c-Met^fl/fl^ and LysCre/c-Met^mut^ mice after chow, MCD (4 weeks), and HFD (24 weeks) feeding. Serum transaminases increase after treatment with steatosis-induced diets (*n* = 8) (^∗^ *p* < 0.05). (c) Representative H&E-stained liver sections of c-Met^fl/fl^ and LysCre/c-Met^mut^ animals (chow, MCD (4 w), and HFD (24 w)) show increased steatosis development in LysCre/c-Met^mut^ mice. Magnification: 200x; scale bars: 100 *μ*m. (d) Representative images of Oil Red O-stained liver sections from c-Met^fl/fl^ and LysCre/c-Met^mut^ mice after chow, MCD (4 weeks), and HFD (24 weeks) treatment are depicted. Magnification: 200x; scale bars: 100 *μ*m. (e) After 24 weeks of HFD feeding, LysCre/c-Met^mut^ animals displayed a significantly impaired glucose tolerance shown by a stronger increase in serum glucose levels compared to equally treated c-Met^fl/fl^ mice (*n* = 8) (^∗^ *p* < 0.05). (f) Intrahepatic triglyceride levels were determined in livers of chow, MCD (4 weeks), or HFD (24 weeks) fed c-Met^fl/fl^ and LysCre/c-Met^mut^ mice. At least 5 animals per group were included (^∗^ *p* < 0.05).

To evaluate the significance of c-Met in Kupffer cells/myeloid cells for steatohepatitis development, 8–12-week-old c-Met^fl/fl^ and LysCre/c-Met^mut^ littermates were fed either chow, methionine-choline-deficient diet (MCD) (4 weeks), or high-fat diet (HFD) (24 weeks) *ad libitum*. A significant increase in serum transaminase levels in LysCre/c-Met^mut^ animals as well as in the MCD and HFD models points to a severe disease progression of nonalcoholic steatohepatitis (NASH) (Figures 1(a) and 1(b)). This invasive disease development was further reflected in faster progressing steatosis directly associated with detrimental changes in liver architecture and fatty liver degeneration which is shown in the histological analysis (H&E Figure 1(c), Oil Red O Figure 1(d)). To investigate metabolic changes over 24 weeks of treatment with steatosis-induced diets, we next performed a glucose tolerance test to detect typical features of the metabolic syndrome. Significantly elevated serum glucose levels in LysCre/c-Met^mut^ mice compared to equally fed c-Met^fl/fl^ animals indicated the existence of insulin resistance (Figure 1(e)). To directly measure the hepatic triglyceride accumulation as a hallmark of nonalcoholic fatty liver disease, homogenates of whole liver extracts of c-Met^fl/fl^ and LysCre/c-Met^mut^ animals were assessed regarding their triglyceride content after chow, MCD (4 weeks), and HFD (24 weeks) feeding. After treatment with both steatohepatitis-induced diets, LysCre/c-Met^mut^ mice displayed a significantly higher accumulation of hepatic triglycerides compared to c-Met^fl/fl^ controls (Figure 1(f)). The data suggests a protective role of c-Met in Kupffer cells in the development of diet-induced steatosis.

To further assess the progression from simple steatosis to a more advanced disease state of steatohepatitis, we next investigated whether the imbalance in systemic glucose and lipid metabolism resulted in alterations in the immune cell response.

**Figure 2 fig2:**
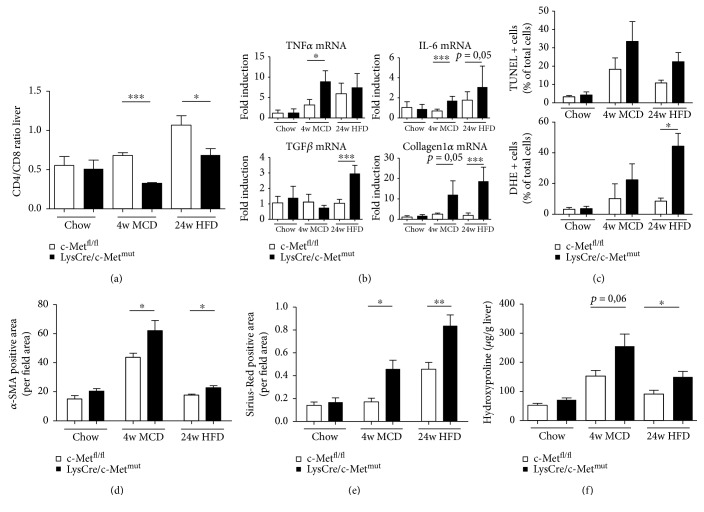
Stronger proinflammatory immune response and fibrosis development in livers of LysCre/c-Met^mut^ animals. (a) Intrahepatic CD4^+^ and CD8^+^ T cells were analyzed by flow cytometry after chow, 4 weeks of MCD, or 24 weeks of HFD feeding of c-Met^fl/fl^ and LysCre/c-Met^mut^ mice. CD4^+^ and CD8^+^ T cells were gated by FSC/SSC (duplets were excluded), live/CD45^+^, CD4^+^_,_ or CD8^+^. A statistical analysis of the ratio of CD4^+^/CD8^+^ T cells of recorded cells was performed (*n* = 5) (^∗^ *p* < 0.05, ^∗∗∗^ *p* < 0.001). (b) mRNA expression levels of TNF-*α*, IL-6, TGF-*β*, and Collagen1*α*. Whole liver homogenates of c-Met^fl/fl^ or LysCre/c-Met^mut^ mice were analyzed via real-time PCR. The quantification is expressed as fold induction over the mean values obtained for chow fed c-Met^fl/fl^ livers. At least 6 animals per group were included (^∗^ *p* < 0.05, ^∗∗∗^ *p* < 0.001). (c) Statistical analysis of the percentage of TUNEL^+^ and DHE^+^ cells referred to the number of total cells on stained liver sections of c-Met^fl/fl^ and LysCre/c-Met^mut^ mice treated either with chow or steatohepatitis-induced diets. 10 view fields/liver of at least *n* = 4 animals per genotype and time point were included (scale bars: 100 *μ*m; magnification: 200x) (^∗^ *p* < 0.05). (d) Quantitative analysis of *α*-SMA-stained liver cryosections of c-Met^fl/fl^ and LysCre/c-Met^mut^ mice after chow, MCD (4 weeks), or HFD (24 weeks) feeding. Scale bars: 100 *μ*m; magnification: 200x. Quantitative analysis of *α*-SMA staining was calculated by evaluating the *α*-SMA^+^ area per field area of 10 view fields/liver by ImageJ© (^∗^ *p* < 0.05). *n* = 4 animals per group and genotype were included. (e) For quantitative analysis of Sirius Red staining, the Sirius Red-positive area per view field of 10 view fields/liver of chow, MCD (4 weeks), or HFD (24 weeks) fed c-Met^fl/fl^ or LysCre/c-Met^mut^ animals was analyzed and recorded under polarized light by ImageJ© (^∗^ *p* < 0.05, ^∗∗^ *p* < 0.01). Included were at least *n* = 4 animals per genotype and time point. (f) Displayed are hydroxyproline levels of chow, MCD (4 weeks), or HFD (24 weeks) fed c-Met^fl/fl^ and LysCre/c-Met^mut^ mice (^∗^ *p* < 0.05) (*n* = 4).

Flow cytometric analysis of the intrahepatic immune cell infiltration revealed a decrease in the ratio of CD4^+^/CD8^+^ T cells (Figure 2(a), Supplementary Figure 2) which reflects a more dominant CD8^+^ lymphocyte-driven immune cell response in LysCre/c-Met^mut^ animals after MCD and HFD feeding compared to c-Met^fl/fl^ mice. CD8^+^ T cells exert several effector functions including the production of inflammatory cytokines and cytolysis. To investigate this in more detail real-time PCR analysis showed an increase in the mRNA expression of the proinflammatory mediators TNF-*α* and IL-6 and fibrosis markers, such as TGF-*β* and Collagen1*α* in LysCre/c-Met^mut^ after both dietary treatments (Figure 2(b)). TNF-*α* is strongly expressed in animals treated with MCD compared to HFD feeding where it shows only a slight trend to be upregulated. This difference potentially occurs because the MCD model is a non-obesity-related steatohepatitis mouse model with strong inflammatory changes within the liver tissue. TGF-*β* on the contrary is strongly expressed in HFD-treated animals compared to MCD fed LysCre/c-Met^mut^ mice. TGF-*β* is known to be involved in lipid accumulation in hepatocytes in the course of the metabolic syndrome which is more pronounced in the chronic HFD model of murine steatohepatitis compared to MCD feeding [29, 30].

To unravel a potential mechanism responsible for the observed differences in disease development and progression of NASH in mice lacking c-Met in Kupffer cells, we next investigated the amount of apoptotic cell death and the intrahepatic oxidative stress environment by TUNEL and DHE (dihydroethidium (hydroethidine)) staining (Figure 2(c), Supplementary Figures 3A and 3B). LysCre/c-Met^mut^ mice display a trend to an increase in TUNEL-positive and DHE-positive cells after MCD and HFD feeding. An increase in oxidative stress is known to be present in the hepatic microenvironment of livers with NAFLD and NASH which is in turn known to induce the recruitment and infiltration of proinflammatory cells. In particular, T cells are critical in this regard for influencing steatohepatitis development and progression.

Our results thus indicate that the proinflammatory immune response provoked by oxidative stress may be mechanistically at least in parts responsible for the more invasive phenotype in mice with a c-Met deficiency in Kupffer cells.

### 3.3. c-Met Deletion in Kupffer Cells/Myeloid Cells Promotes Fibrosis Progression in Two Dietary Models of Steatohepatitis (MCD, HFD)

A crucial part in the cause of NASH is the progression to fibrosis. To assess this increase in extracellular matrix accumulation, we first performed an immunofluorescence staining for *α*-SMA which revealed a significant increase in LysCre/c-Met^mut^ mice after MCD and HFD feeding compared to equally treated c-Met^fl/fl^ animals (Figure 2(d), Supplementary Figure 3C). An additional Sirius Red staining and the direct measurement of the intrahepatic hydroxyproline content support these results both showing a significant increase in matrix deposition in diet-treated LysCre/c-Met^mut^ mice (Figures 2(e) and 2(f), Supplementary Figure 3D). These results indicate strong hepatic stellate cell activation reflecting fibrosis initiation in animals deficient of c-Met in Kupffer cells.

### 3.4. c-Met Deficiency under the Glial Fibrillary Acid Protein (GFAP) Promoter Leads to Steatosis and Fibrosis

**Figure 3 fig3:**
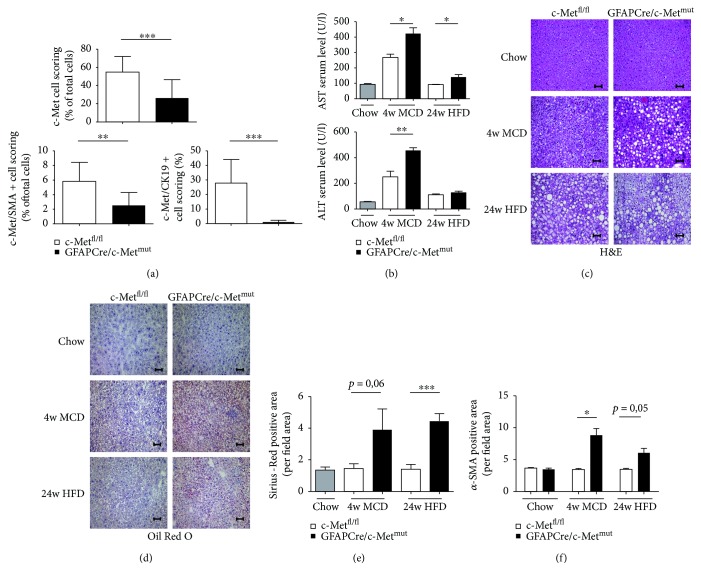
More pronounced disease progression of diet-induced steatohepatitis in GFAPCre/c-Met^mut^ animals. (a) Quantitative evaluation of a multiplex staining approach with simultaneous staining of DAPI, c-Met, *α*-SMA, and CK19 of untreated c-Met^fl/fl^ and GFAPCre/c-Met^mut^ animals. Statistical analysis of the percentage of c-Met single positive cells, c-Met/*α*-SMA double positive cells, and c-Met/CK19 double positive cells referred to the number of total cells is depicted. At least 10 view fields/liver were included at a magnification of 200x (^∗∗^ *p* < 0.01, ^∗∗∗^ *p* < 0.001). (b) Serum transaminase levels (AST and ALT) of c-Met^fl/fl^ and GFAPCre/c-Met^mut^ mice were analyzed after chow, MCD (4 weeks), and HFD (24 weeks) feeding. Serum transaminases increase after treatment with steatohepatitis-induced diets (*n* = 5) (^∗^ *p* < 0.05, ^∗∗^ *p* < 0.01). (c) H&E-stained liver sections of c-Met^fl/fl^ and GFAPCre/c-Met^mut^ animals (chow, MCD (4 w), and HFD (24 w)) show an increase in lipid droplets in GFAPCre/c-Met^mut^ mice. Magnification: 200x; scale bars: 100 *μ*m. (d) Representative images of Oil Red O-stained liver sections from c-Met^fl/fl^ or GFAPCre/c-Met^mut^ mice after chow, MCD (4 weeks), and HFD (24 weeks) treatment are depicted. Magnification: 200x; scale bars: 100 *μ*m. (e) Quantitative analysis of Sirius Red staining of c-Met^fl/fl^ and GFAPCre/c-Met^mut^ mice after chow, 4 weeks of MCD, or 24 weeks of HFD diet. The Sirius Red-positive area was calculated per view field of 10 view fields/liver by ImageJ© (^∗∗∗^ *p* < 0.001). At least *n* = 4 animals per group and genotype were included. (f) Quantitative analysis of *α*-SMA-stained liver cryosections of c-Met^fl/fl^ and GFAPCre/c-Met^mut^ mice after chow, MCD (4 weeks), or HFD (24 weeks) feeding. Scale bars: 100 *μ*m; magnification: 200x. Quantitative analysis of *α*-SMA staining was calculated by evaluating the *α*-SMA^+^ area per field area of 10 view fields/liver by ImageJ© (^∗^ *p* < 0.05). *n* = 4 animals per group and genotype were included.

In the next experiment, it should be investigated which cell types are affected by the deletion of c-Met under the GFAP promoter. In the literature, GFAP is described to be primarily expressed on neural glia cells, especially astrocytes, and on hepatic stellate cells (HSCs) [31]. A multiplex immunofluorescence staining approach for DAPI, c-Met, *α*-SMA (activated hepatic stellate cells), and CK19 (cells of the hepatobiliary tract [32]) in c-Met^fl/fl^ and GFAPCre/c-Met^mut^ animals revealed detailed insight on the targeted cell types and the knockout efficiency (Supplementary Figure 4). Multispectral image analysis with a pattern recognition learning algorithm combined with a trained cell classifier tool identified a c-Met deficiency in GFAPCre/c-Met^mut^ mice, especially in *α*-SMA^+^ and CK19^+^ cells (Figure 3(a), Supplementary Figures 5 and 6). Further analysis of the inflammatory status of these mice under steatohepatitis-induced diets (MCD, HFD) showed a protective effect of c-Met in the targeted liver cells (*α*-SMA^+^/CK19^+^ cells) which gets evident by significantly increased serum transaminase levels in GFAPCre/c-Met^mut^ animals compared to equally treated c-Met^fl/fl^ controls (Figure 3(b)). Histological analysis (H&E, Oil Red O staining) further demonstrate ballooned hepatocytes and fine and coarse fat droplet formation in c-Met^fl/fl^ animals which is more pronounced in the GFAPCre/c-Met^mut^ after MCD (4 weeks) and HFD (24 weeks) feeding (Figures 3(c) and 3(d)). To assess the effect of a c-Met knockout under the GFAP promoter especially in *α*-SMA^+^ cells, we next performed a Sirius Red staining showing a stronger progressing fibrosis development in GFAPCre/c-Met^mut^ mice compared to c-Met^fl/fl^ controls in both dietary models (Figure 3(e), Supplementary Figure 7A). In line with these results, immunofluorescence staining for *α*-SMA reflected stronger fibrosis initiation in GFAPCre/c-Met^mut^ animals after MCD treatment (Figure 3(f), Supplementary Figure 7B).

The data suggest that a deficiency of c-Met in different liver cell types expressing the GFAP promoter leads to a worsened disease outcome in two independent mouse models of steatohepatitis.

### 3.5. Deletion of c-Met in Bone Marrow-Derived Immune Cells Leads to Severe Development and Progression of Steatohepatitis

**Figure 4 fig4:**
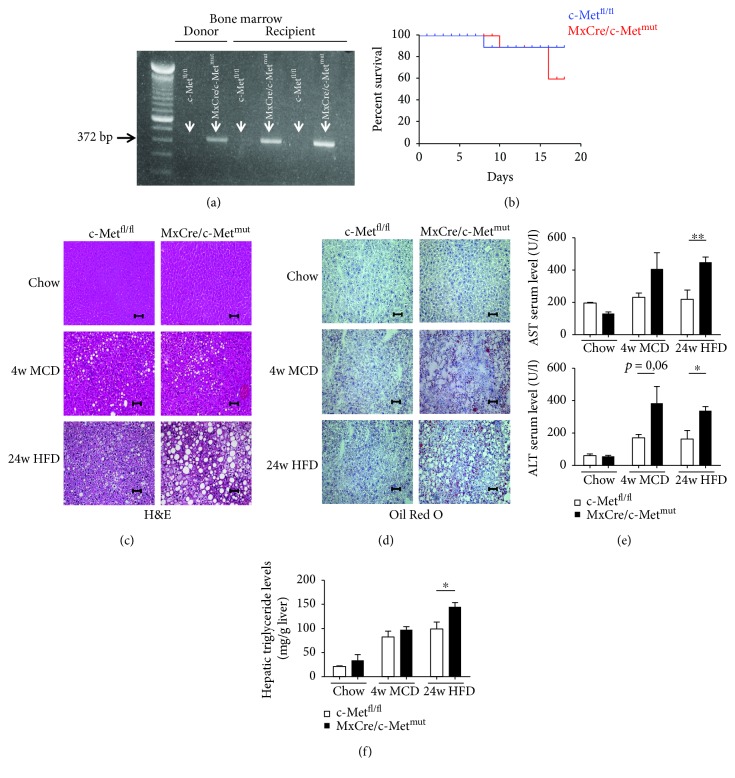
Earlier onset of steatosis in MxCre/c-Met^mut^ mice after MCD or HFD feeding. (a) Control PCR for the knockout PCR product of c-Met^fl/fl^ and MxCre/c-Met^mut^ mice. DNA of isolated bone marrow after bone marrow transplantation was used as template. The 372 bp product represents the MxCre/c-Met^mut^ allele. (b) After bone marrow transplantation, it was particularly noticed that nearly 50% MxCre/c-Met^mut^ mice died within 16 days. Therefore, survival curves were calculated for c-Met^fl/fl^ and MxCre/c-Met^mut^ animals after bone marrow transplantation. (c) Representative H&E-stained liver sections of c-Met^fl/fl^ and MxCre/c-Met^mut^ animals (chow, MCD (4 w), and HFD (24 w)) show an increase in steatosis development in MxCre/c-Met^mut^ mice after treatment with steatohepatitis-induced diets. Magnification: 200x; scale bars: 100 *μ*m. (d) Oil Red O-stained liver sections from c-Met^fl/fl^ and MxCre/c-Met^mut^ mice after chow, MCD (4 weeks), and HFD (24 weeks) treatment are depicted. Magnification: 200x; scale bars: 100 *μ*m. (e) AST and ALT serum levels of c-Met^fl/fl^ and MxCre/c-Met^mut^ mice after chow, 4 weeks of MCD, and 24 weeks of HFD feeding. Serum transaminases increase after treatment with chronic liver injury-induced diets (*n* = 6) (^∗^ *p* < 0.05, ^∗∗^ *p* < 0.01). (f) Intrahepatic triglyceride levels were determined in livers of chow, MCD (4 weeks), or HFD (24 weeks) fed c-Met^fl/fl^ and MxCre/c-Met^mut^ mice. At least 6 animals per group were included (^∗^ *p* < 0.05).

**Figure 5:**
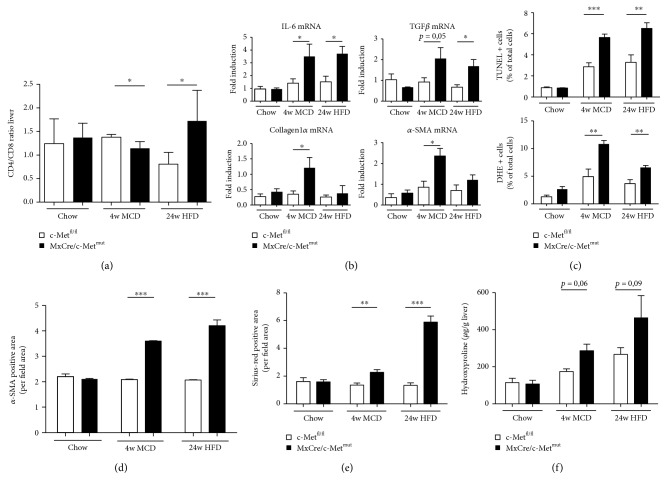
Loss of c-Met on MxCre^+^ cells strengthens the proinflammatory immune response after chronic liver injury. (a) Intrahepatic CD4^+^ and CD8^+^ T cells were analyzed by flow cytometry after chow, 4 weeks of MCD, or 24 weeks of HFD feeding of c-Met^fl/fl^ and MxCre/c-Met^mut^ mice. CD4^+^ and CD8^+^ T cells were gated by FSC/SSC (duplets were excluded), live/CD45^+^, CD4^+^, or CD8^+^. A statistical analysis of the ratio of CD4^+^/CD8^+^ T cells of recorded cells was performed (*n* = 5) (^∗^ *p* < 0.05). (b) mRNA expression levels of IL-6, TGF-*β*, Collagen1*α*, and *α*-SMA. Whole liver homogenates of c-Met^fl/fl^ or MxCre/c-Met^mut^ mice were analyzed via real-time PCR. The quantification is expressed as fold induction over the mean values obtained for chow fed c-Met^fl/fl^ livers. At least 5 animals per group were included (^∗^ *p* < 0.05). (c) Statistical analysis of the percentage of TUNEL^+^ and DHE^+^ cells referred to the total number of cells on stained liver sections of c-Met^fl/fl^ and MxCre/c-Met^mut^ mice treated either with chow or steatohepatitis-induced diets. 10 view fields/liver of at least *n* = 5 animals per genotype and time point were included (scale bars: 100 *μ*m; magnification: 200x) (^∗∗^ *p* < 0.01, ^∗∗∗^ *p* < 0.001). (d) Quantitative analysis of *α*-SMA-stained liver cryosections of c-Met^fl/fl^ and MxCre/c-Met^mut^ mice after chow, MCD (4 weeks), or HFD (24 weeks) feeding. Scale bars: 100 *μ*m; magnification: 200x. Quantitative analysis of *α*-SMA staining was calculated by evaluating the *α*-SMA^+^ area per field area of 10 view fields/liver by ImageJ© (^∗^ *p* < 0.05). *n* = 4 animals per group and genotype were included. (e) For quantitative analysis of Sirius Red staining, the Sirius Red-positive area per view field of 10 view fields/liver of chow, MCD (4 weeks), or HFD (24 weeks) fed c-Met^fl/fl^ or MxCre/c-Met^mut^ animals was analyzed and recorded under polarized light by ImageJ© (^∗∗^ *p* < 0.01, ^∗∗∗^ *p* < 0.001). Included were at least *n* = 5 animals per genotype and time point. (f) Hydroxyproline levels of chow, MCD (4 weeks), or HFD (24 weeks) fed c-Met^fl/fl^ and MxCre/c-Met^mut^ mice are displayed (*n* = 5).

Next, we investigated a targeted c-Met deletion in hematopoietic cells under the inducible (Mx dynamin-like GTPase Cre) MxCre promoter. After bone marrow transplantation, the efficiency of the c-Met deletion was investigated by a knockout PCR approach. The 372 bp PCR product shows the effective deletion of c-Met either in the donor or in the associated recipient (Figure 4(a)). Surprisingly, c-Met deficiency in hematopoietic cells (MxCre/c-Met^mut^) resulted in severe maintenance of survival compared to controls (c-Met^fl/fl^) (Figure 4(b)). In support of the idea that c-Met is protective in different liver cell types, histological analysis of H&E and Oil Red O staining again displayed massive fatty liver degeneration and earlier and stronger signs of steatosis in MxCre/c-Met^mut^ animals after MCD and HFD treatment compared to floxed controls (c-Met^fl/fl^) (Figures 4(c) and 4(d)). Representative images illustrated more ballooned hepatocytes and fine and coarse dropping steatosis in mice deficient of c-Met in hematopoietic cells whereas c-Met^fl/fl^ mice showed fewer hepatocyte ballooning and just fine fat droplet formation. The increase in steatosis development was further reflected by slightly increased serum aspartate aminotransferase (AST) and alanine aminotransferase (ALT) levels (Figure 4(e)) and higher hepatic triglyceride levels especially after 24-week HFD treatment (Figure 4(f)) in MxCre/c-Met^mut^ compared to c-Met^fl/fl^ mice. To specifically investigate whether the worsened disease outcome was associated with stronger immune cell infiltration, flow cytometric analysis was performed. As hepatic inflammation is one of the main factors differentiating simple steatosis from progressive steatohepatitis, the investigation of the hepatic infiltration of CD4^+^ and CD8^+^ T cells showed significant differences in the ratio of these cell types. After 4 weeks of MCD treatment, a significant shift to a more dominant CD8^+^ T cell response was evident in MxCre/c-Met^mut^ animals. On the contrary, after 24-week HFD feeding, the T cell response reverses to a CD4^+^ cell-prone immune response in the MxCre/c-Met^mut^ (Figure 5(a)). To test whether this contributes to a pro- or anti-inflammatory cytokine profile, we next determined the mRNA expression level of IL-6 which was significantly upregulated in MxCre/c-Met^mut^ in both treatment groups compared to c-Met^fl/fl^ controls (Figure 5(b)).

To elucidate the disparity of steatohepatitis development and manifestation between controls and mice lacking c-Met on hematopoietic cells, we tried to further characterize potential underlying mechanisms in more detail. In animals with a specific deletion of c-Met in Kupffer cells, we previously observed differences in the oxidative stress milieu which may trigger hepatic inflammation in the two dietary mouse models of steatohepatitis (MCD, HFD). To investigate whether this is a general phenomenon when c-Met signaling is blocked, we also performed TUNEL and DHE staining in c-Met^fl/fl^ and MxCre/c-Met^mut^ mice after chow, MCD, or HFD feeding. Comparable to what was found in c-Met^fl/fl^ and LysCre/c-Met^mut^ animals, mice deficient of c-Met in hematopoietic cells displayed a significantly greater amount of TUNEL- and DHE-positive cells whereas no difference in the expression of the antioxidative regulator Nrf2 could be detected after MCD and HFD treatment (Figure 5(c), Supplementary Figures 8A, 8B, and 8C). Collectively, these data suggest a general modulatory effect of c-Met on any intrahepatic cell on the oxidative microenvironment associated with exacerbated steatohepatitis.

### 3.6. Stronger Fibrosis Progression in MxCre/c-Met^mut^ Mice after HFD and MCD Feeding

The progression from fatty liver degeneration to the excessive accumulation of extracellular matrix within liver tissue reflects the typical course of NASH disease development. To assess the progression towards fibrosis, the mRNA expression of TGF-*β*, Collagen1a1, and *α*-SMA was measured via real-time PCR. All investigated markers showed increases in MxCre/c-Met^mut^ animals compared to c-Met^fl/fl^ controls after dietary treatments whereas the increase was clearly more pronounced in MCD fed mice compared to HFD fed mice. HFD-treated animals displayed only a minor but visible upregulation (Figure 5(b)). In addition, we found a significant increase in intrahepatic *α*-SMA levels (Figure 5(d), Supplementary Figure 9A), Sirius Red-positive collagen fibers (Figure 5(e), Supplementary Figure 9B), and a moderately upregulated hydroxyproline content (Figure 5(f)) in MxCre/c-Met^mut^ mice after MCD and HFD.

Taken together, the data clearly shows that c-Met in Kupffer cells/macrophages, *α*-SMA^+^/CK19^+^ cells, and bone marrow-derived immune cells is involved in pushing the intrahepatic microenvironment to more oxidative stress finally leading to severe development of steatohepatitis and the progression to fibrosis.

### 3.7. c-Met Is Increased in Human Cirrhotic Patients but Drops in NASH Patients

**Figure 6:**
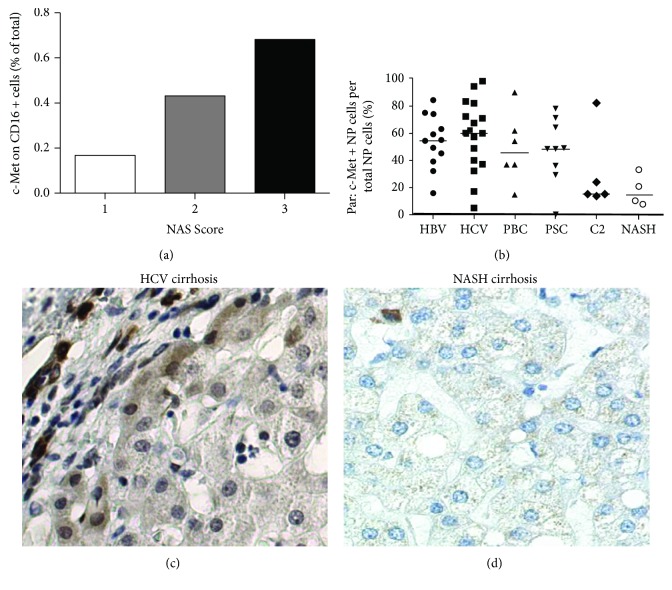
c-Met expression is decreased in NASH patients. (a) c-Met expression of intrahepatic nonclassical human monocytes analyzed by flow cytometry correlated with the NAFLD activity score (NAS). (b) c-Met expression on nonparenchymal (NP) cells of patients with HBV (hepatitis B virus), HCV (hepatitis C virus), PBC (primary biliary cholangitis), PSC (primary sclerosing cholangitis), C2 (alcoholic) cirrhosis, and NASH was analyzed by immunohistochemistry. Immunohistochemical staining of c-Met on liver sections of patients with (c) HCV-related cirrhosis and (d) NASH cirrhosis.

In two different mouse models of diet-induced steatohepatitis, we found strong development of nonalcoholic steatohepatitis and progression to fibrosis when c-Met is missing in any liver cell type. To confirm the dominant role of c-Met on nonclassical monocytes during fibrosis development, we correlated the expression of c-Met on CD16^+^ immune cells with the NAFLD activity score (NAS) of patient samples. The correlation clearly demonstrates a decrease in c-Met expression with less severe disease development (Figure 6(a)). Supporting this data, immunohistochemistry analysis of human patient samples showed strong c-Met expression in patients suffering from HBV (hepatitis B virus), HCV (hepatitis C virus), PBC (primary biliary cholangitis), and PSC (primary sclerosing cholangitis). However, patients with alcoholic cirrhosis and NASH patients display decreased c-Met levels (Figure 6(b)). In addition to this, immunohistochemical staining of c-Met in human samples reflected a strong c-Met expression in patients with HVC-related cirrhosis (Figure 6(c)) and nearly no c-Met expression in NASH patients (Figure 6(d)).

## 4. Discussion

The first transgenic mouse experiments with c-Met were performed in 1996 by Liang and colleagues [33]. In this study, mice exhibited a constitutive activation of the c-Met protein caused by constant dimerization. This overactivation leads to the spontaneous development of mammary hyperplasia and tumors, as a direct effect of the transgene at 6–9 months of age. Also, in primary tumors of human patient samples (liver metastasis from colon carcinoma [34], non-small-cell lung carcinoma [35, 36]), c-Met overexpression and constitutive kinase activity were present. Mechanistically, it could be shown that the overexpression of c-Met is driven by hypoxia in the center of the growing tumor [37]. On the contrary, it could be shown that HGF, the only known ligand for c-Met, conveys protective effects in different chronic diseases such as liver cirrhosis and lung fibrosis [38–40].

In the previous study, our group could show that c-Met plays a pivotal role during the development of NASH in a hepatocyte-specific knockout mouse model [41]. Nevertheless, the contribution of c-Met on other liver cell types and on fibrosis initiation besides hepatocytes is only poorly understood.

Therefore, the aim of the present study was to investigate the significance of c-Met on different intrahepatic and infiltrating cell types in the initiation and progression of NASH-related fibrosis. Ueki et al. could show in 1999 that the activation of the HGF/c-Met signaling pathway decreases TGF-*β*1 levels. Based on these findings, they proposed HGF/c-Met gene therapy for the treatment of patients with liver cirrhosis.

We here hypothesized that c-Met prevents from NASH-related fibrosis and wanted to clarify in detail on which cell type (LysCre for Kupffer cells/macrophages, GFAPCre for *α*-SMA^+^ and CK19^+^ cells, and MxCre for bone marrow-derived immune cells) c-Met is essentially involved in this process.

A large number of experiments in different inflammatory diseases show controversial effects of the activation of the HGF/c-Met axis. In atherosclerosis, HGF was found to be associated with disease progression [42], whereas in a pancreatitis model in mice and in drug-induced oxidative liver damage [43], results displayed protective effects of the administration of recombinant human HGF.

As hepatic manifestation of the metabolic syndrome, the development of NASH is associated with an impaired insulin metabolism. Varkaris et al. could show in 2013 that the insulin-like growth factor-1 (IGF-1) signaling through the IGF-1 receptor is able to delay the induction of c-Met activation, thereby showing a direct interaction between the HGF/c-Met signaling axis with metabolic changes as they can occur in steatohepatitis [44]. Thus, strengthening the hypothesis of a direct involvement of c-Met in diet-induced NASH fibrosis. In this study, the loss of c-Met on different liver cell types (Kupffer cells/macrophages, *α*-SMA^+^/CK19^+^ cells, and bone marrow-derived immune cells) led to severe steatosis, steatohepatitis, and fibrosis progression in two different mouse models of diet-induced steatohepatitis in all three used Cre lines (Figures 1 [Fig fig2] [Fig fig3] [Fig fig4]–5). Further, human patients with NASH show nearly no c-Met expression in liver tissue (Figure 6). These results support the idea that c-Met protects from fibrosis. Strong fat droplet accumulation and the more pronounced inflammatory response are further associated with an increased number of apoptotic cells together with a strong activation of the oxidative stress response in livers of mice with a cell-specific c-Met deficiency after treatment with NASH-induced diets. Xiao et al. could show in 2001 that c-Met signals via AKT/protein kinase B are primarily responsible for cell survival [45]. Additionally, previous work of our group further demonstrated that HGF/c-Met actively regulates hepatocyte differentiation [46]. Both studies support the finding that the loss of c-Met on different liver cells (Kupffer cells/macrophages, *α*-SMA^+^/CK19^+^ cells, and bone marrow-derived immune cells) leads to an increase in the oxidative stress response and apoptotic cell death under dietary treatment.

It is well described that the infiltration of proinflammatory monocytes contributes to NASH progression [47]. The role of HGF/c-Met signaling on liver resident and infiltrating monocytes during NASH development however remains unclear. The loss of c-Met on monocytes expressing the LysMCre promoter leads to a deterioration in the course of liver disease in MCD- as well as in HFD-induced steatohepatitis.

LysCre/c-Met^mut^ animals display earlier fat droplet accumulation, a stronger onset of insulin resistance, and an increase in the proinflammatory immune response finally leading to massive extracellular matrix deposition (Figures 2 and 3). In 2012, Ishikawa et al. could show that c-Met deficiency leads to defects in the mobilization of Kupffer cells in the DDC model of chronic liver injury [48]. Based on this, we hypothesize less stimulation of the anti-inflammatory and antioxidative stress response leading to an increase in apoptosis, oxidative stress, and in the end fibrosis progression.

Previous studies could show that hepatic stellate cells (HSCs) that express the GFAPCre promotor play a major role in the development of liver fibrosis [49]. Under inflammatory conditions, they transdifferentiate into myofibroblast-like cells producing extracellular matrix proteins which accumulate within liver tissue [50]. To which extent the activation of the HGF/c-Met axis is involved in this process is not well understood yet. GFAPCre/c-Met^mut^ mice exhibit severe steatohepatitis development after dietary treatment with increased transaminase levels, histomorphological changes, and the accumulation of extracellular matrix components (Figure 4). Previous studies in our group could show that the loss of c-Met on hepatocytes leads to chronic tissue damage and fibrosis progression in the bile duct ligation model of chronic liver injury [11]. This effect is based on an imbalance between antiapoptotic functions of c-Met and its proproliferative capacity. In c-Met^∆Hepa^ mice, this lead to an increase in the proinflammatory immune response and strong activation of hepatic stellate cells. The same mechanism seems to be active when c-Met is missing on *α*-SMA^+^/CK19^+^ cells directly. Strong activation of the proinflammatory immune response drives an increased activation of hepatic stellate cells and thereby the accumulation of extracellular matrix.

The infiltration of immune cells is one of the characteristic differences between simple steatosis and the more severe steatohepatitis. Recent investigations describe an immunoregulatory function of c-Met on hematopoietic progenitor cells [51, 52]. Further studies additionally show the expression of c-Met also on neutrophils and CD8^+^ T cells upon proinflammatory cytokine exposure during tissue injury [53, 54]. In this study, we examined the role of c-Met on infiltrating bone marrow-derived immune cells to the liver in two dietary mouse models of steatohepatitis. Our experiments clearly show that the loss of c-Met on migrating immune cells carrying the MxCre promotor leads to severe steatosis, inflammation, and fibrosis development (Figures 5 and 6). The recent study of Wang et al. shows that the overexpression of c-Met on bone marrow-derived mesenchymal stem cells leads to an improvement of their homing capacity, their mobilization potential, and their repair function in acute liver failure in rats [55]. Defective migration for its part leads to the absence of the activation of anti-inflammatory immune responses resulting in the observed proinflammatory phenotype. This is further reflected by a significant increase in apoptotic cell death within liver tissue together with massive production of reactive oxygen species (ROS) causing tissue damage and thus fibrosis.

Taken together, our findings surprisingly show that we could find no difference in the degree and outcome of diet-induced steatohepatitis initiation and progression independent on which cell type c-Met is lacking. This indicates a global role of c-Met in a cross talk between intrahepatic cells involved in steatohepatitis development and a protective effect of c-Met in fibrosis generation. Mechanistically, the knockout of c-Met leads to an imbalance of different pro- and anti-inflammatory pathways with an increase in apoptotic cell death together with a strong activation of ROS production which may in part explain the increase in liver injury leading to severe fibrosis progression.

On the contrary, there is a growing quantity of clinical trials, e.g., ficlatuzumab, which is a humanized HGF inhibitory monoclonal antibody preventing HGF/c-Met signaling by blocking the ligand-mediated activation which is actually in a phase 1b clinical trial for the treatment of non-small-cell lung cancer [56]. Further, there is an increasing body of interest in testing different nonselective and selective c-Met inhibitors in the treatment of HCC [26]. Therefore, the role of c-Met signaling in the chronically injured liver seems to be contextual and also tissue specific.

## Data Availability

The data used to support the findings of this study are included within the article and within the supplementary information file.

## Abbreviations

ALT: Alanine aminotransferase
AST: Aspartate aminotransferase
DHE: Dihydroethidium (hydroethidine)
GFAP: Glial fibrillary acid protein
HBV: Hepatitis B virus
HCC: Hepatocellular carcinoma
HCV: Hepatitis C virus
HFD: High-fat diet
HGF: Hepatocyte growth factor
HSC: Hepatic stellate cell
KO: Knockout
MCD: Methionine-choline-deficient diet
NAFLD: Nonalcoholic fatty liver disease
NAS: NAFLD activity score
NASH: Nonalcoholic steatohepatitis
PBC: Primary biliary cholangitis
PSC: Primary sclerosing cholangitis
ROS: Reactive oxygen species
TGF-*β*: Transforming growth factor beta.

## Supplementary Materials

Supplementary Materials Supplementary Figure 1: (A) genomic PCR genotyping using oligos for recombined and nonrecombined alleles. Comparison of knockout PCR products using c-Met^fl/fl^ and LysCre/c-Met^mut^ mouse DNA of isolated primary hepatocytes, *α*-SMA+/CK19+ cells and bone marrow-derived immune cells. GFAPCre/c-Met^mut^ mouse DNA of isolated primary hepatocytes, Kupffer cells/macrophages and bone marrow-derived immune cells. MxCre/c-Met^mut^ mouse DNA of isolated primary hepatocytes, *α*-SMA+/CK19+ cells and Kupffer cells/macrophages. The 500 bp product represents the c-Met^fl/fl^ allele; the 800 bp product shows the c-Met^mut^ genotype. (B) Comparison of knockout PCR products using c-Met^fl/fl^ and LysCre/c-Met^mut^ mouse DNA of isolated primary Kupffer cells/macrophages. The 500 bp product represents the c-Met^fl/fl^ allele; the 800 bp product shows the c-Met^mut^ genotype. (C) Comparison of knockout PCR products using c-Met^fl/fl^ and GFAP/c-Met^mut^ mouse DNA of isolated primary *α*-SMA+/CK19+ cells. The 500 bp product represents the c-Met^fl/fl^ allele; the 800 bp product shows the c-Met^mut^ genotype. (D) Comparison of knockout PCR products using c-Met^fl/fl^ and Mx/c-Met^mut^ mouse DNA of isolated primary bone marrow-derived immune cells. The 500 bp product represents the c-Met^fl/fl^ allele; the 800 bp product shows the c-Met^mut^ genotype. Supplementary Figure 2: representative gating of the analysis of intrahepatic CD4+ and CD8+ T cells in c-Met^fl/fl^ animals after HFD treatment for 24 weeks. Cells are gated FSC/SSC, FSC-W/FSC-A, live dead/CD45+, CD4+, or CD8+. Supplementary Figure 3: representative photographs of (A) TUNEL-, (B) DHE-, (C) *α*-SMA-, and (D) Sirius Red-stained liver sections of c-Met^fl/fl^ and LysCre/c-Met^mut^ mice after chow, MCD (4 weeks), or HFD (24 weeks) feeding. Scale bars: 100 *μ*m; magnification: 200x. Supplementary Figure 4: images of multiplexed immunofluorescence-stained liver sections (DAPI, c-Met, alpha-SMA, and CK19) of c-Met^fl/fl^ and GFAPCre/c-Met^mut^ animals. Magnification: 200x and 400x. Together with the corresponding images of the single channels of the multiplexed immunofluorescence-stained liver sections (DAPI, c-Met, alpha-SMA, and CK19) of c-Met^fl/fl^ and GFAPCre/c-Met^mut^ mice. Magnification: 400x. Supplementary Figure 5: scans of the multispectral image acquisition and analysis showing the raw image, cell segmentation, phenotyping, and scoring of a c-Met^fl/fl^ and GFAPCre/c-Met^mut^ mouse. Supplementary Figure 6: (A) 2D and (B) 3D scattered plots based on the analysis of the mean fluorescent intensity of c-Met^fl/fl^ and GFAPCre/c-Met^mut^ animals with MATLAB R2016b (MathWorks, Natick, USA). Supplementary Figure 7: representative images of (A) Sirius Red-stained liver sections and (B) of *α*-SMA-stained liver sections of c-Met^fl/fl^ and GFAPCre/c-Met^mut^ mice after dietary treatment (chow, MCD 6 weeks, and HFD 24 weeks). Scale bars: 100 *μ*m; magnification: 200x. Supplementary Figure 8: representative photographs of (A) TUNEL-, (B) DHE-, and (C) Nrf2-stained liver sections of c-Met^fl/fl^ and MxCre/c-Met^mut^ mice after chow, MCD (4 weeks), or HFD (24 weeks) feeding. Scale bars: 100 *μ*m; magnification: 200x. Supplementary Figure 9: representative photographs of (A) *α*-SMA- and (B) Sirius Red-stained liver sections of c-Met^fl/fl^ and MxCre/c-Met^mut^ mice after chow, MCD (4 weeks), or HFD (24 weeks) feeding. Scale bars: 100 *μ*m; magnification: 200x.
