# Supplementary material for: c-Met Signaling Protects from Nonalcoholic Steatohepatitis- (NASH-) Induced Fibrosis in Different Liver Cell Types
Source: Oxid Med Cell Longev. 2018 Nov 12;2018:6957497. doi: 10.1155/2018/6957497 (PMC6260421; doi:10.1155/2018/6957497)
Supplement: Supplementary Materials — Supplementary Figure 1: (A) genomic PCR genotyping using oligos for recombined and nonrecombined alleles. Comparison of knockout PCR products using c-Metfl/fl and LysCre/c-Metmut mouse DNA of isolated primary hepatocytes, α-SMA+/CK19+ cells and bone marrow-derived immune cells. GFAPCre/c-Metmut mouse DNA of isolated primary hepatocytes, Kupffer cells/macrophages and bone marrow-derived immune cells. MxCre/c-Metmut mouse DNA of isolated primary hepatocytes, α-SMA+/CK19+ cells and Kupffer cells/macrophages. The 500 bp product represents the c-Metfl/fl allele; the 800 bp product shows the c-Metmut genotype. (B) Comparison of knockout PCR products using c-Metfl/fl and LysCre/c-Metmut mouse DNA of isolated primary Kupffer cells/macrophages. The 500 bp product represents the c-Metfl/fl allele; the 800 bp product shows the c-Metmut genotype. (C) Comparison of knockout PCR products using c-Metfl/fl and GFAP/c-Metmut mouse DNA of isolated primary α-SMA+/CK19+ cells. The 500 bp product represents the c-Metfl/fl allele; the 800 bp product shows the c-Metmut genotype. (D) Comparison of knockout PCR products using c-Metfl/fl and Mx/c-Metmut mouse DNA of isolated primary bone marrow-derived immune cells. The 500 bp product represents the c-Metfl/fl allele; the 800 bp product shows the c-Metmut genotype. Supplementary Figure 2: representative gating of the analysis of intrahepatic CD4+ and CD8+ T cells in c-Metfl/fl animals after HFD treatment for 24 weeks. Cells are gated FSC/SSC, FSC-W/FSC-A, live dead/CD45+, CD4+, or CD8+. Supplementary Figure 3: representative photographs of (A) TUNEL-, (B) DHE-, (C) α-SMA-, and (D) Sirius Red-stained liver sections of c-Metfl/fl and LysCre/c-Metmut mice after chow, MCD (4 weeks), or HFD (24 weeks) feeding. Scale bars: 100 μm; magnification: 200x. Supplementary Figure 4: images of multiplexed immunofluorescence-stained liver sections (DAPI, c-Met, alpha-SMA, and CK19) of c-Metfl/fl and GFAPCre/c-Metmut animals. Magnification: 200x and 400x. To [file 6957497.f1.pdf]

Supplementary Figure 1

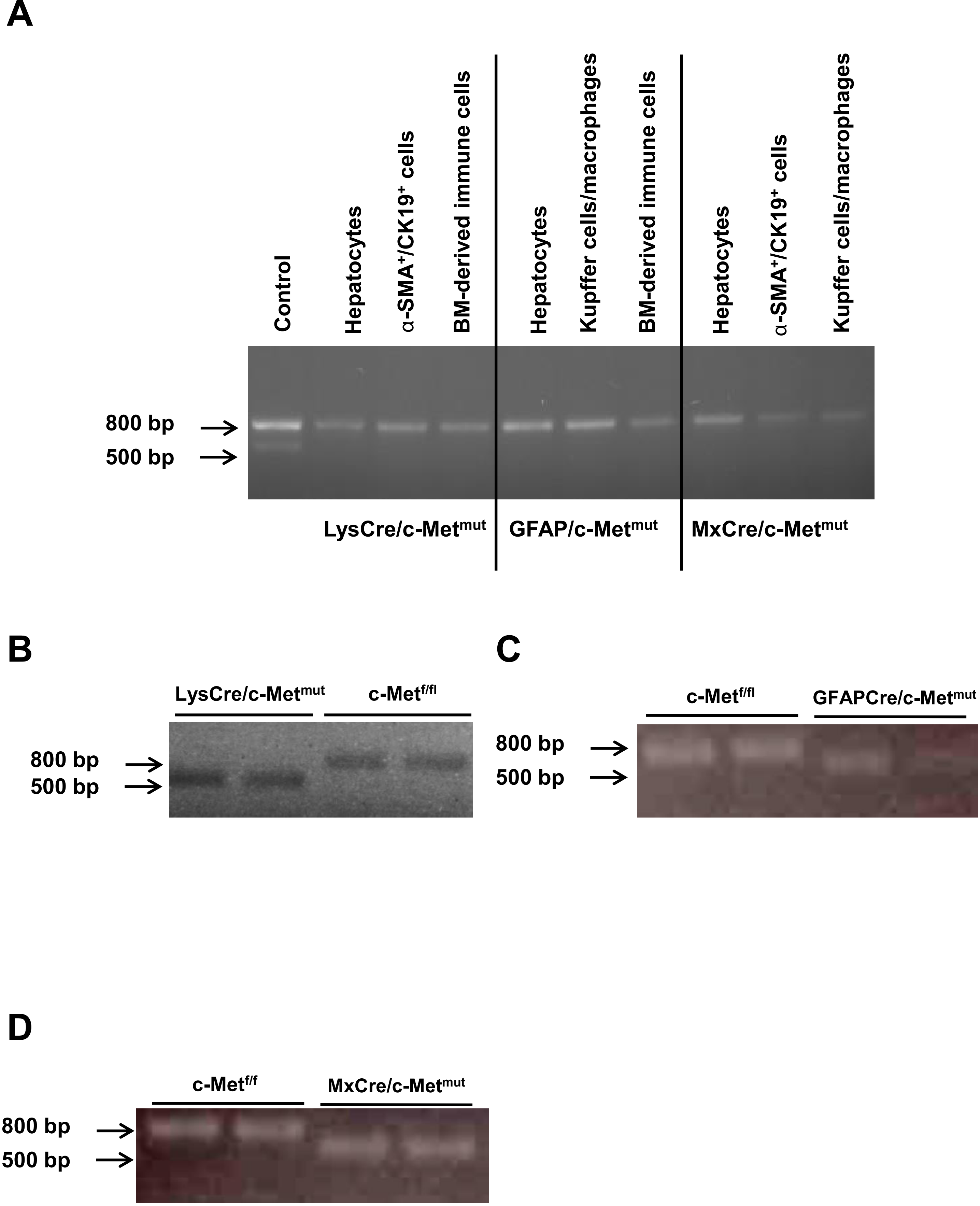

Supplementary Figure 2

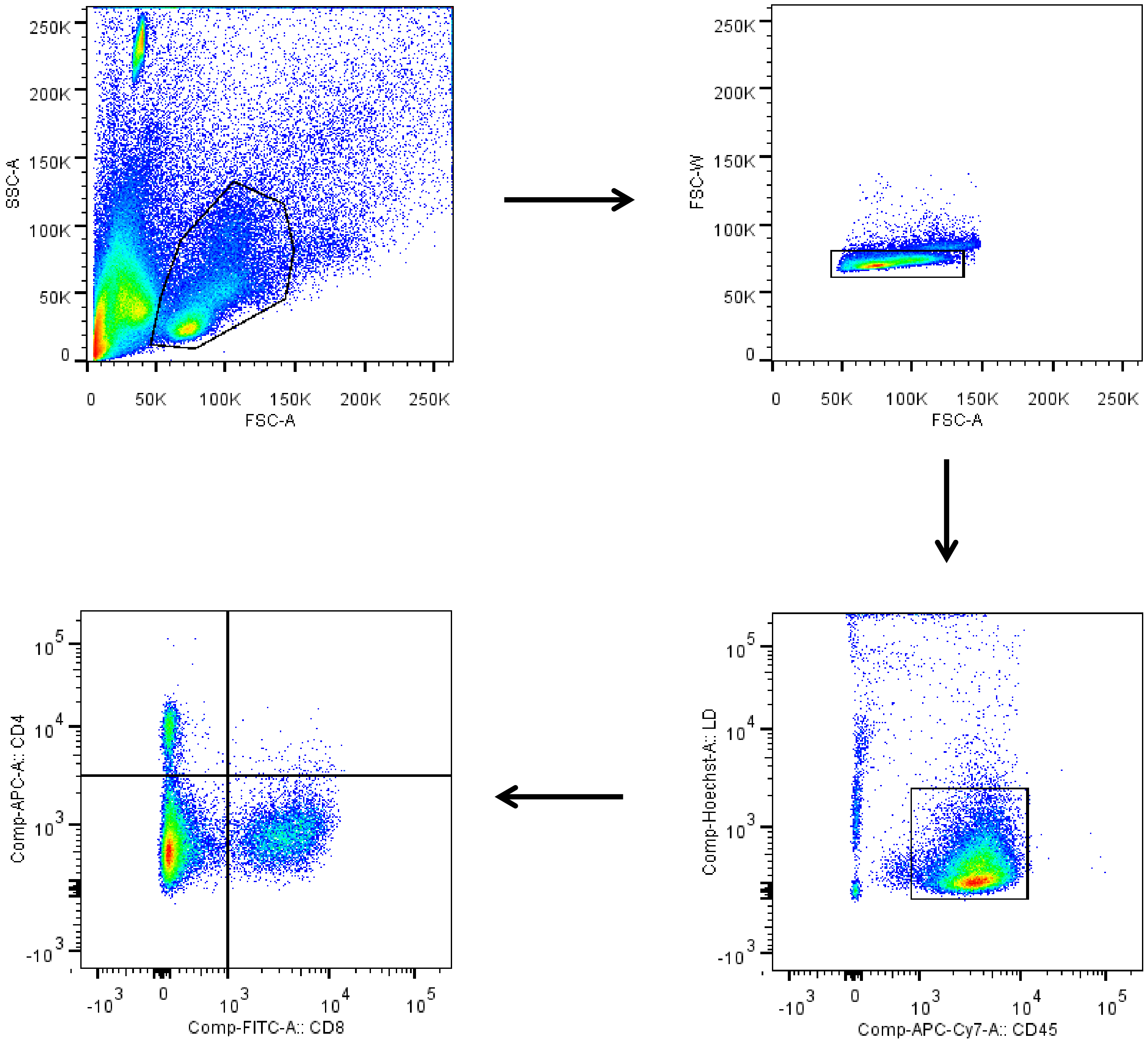

Supplementary Figure 3

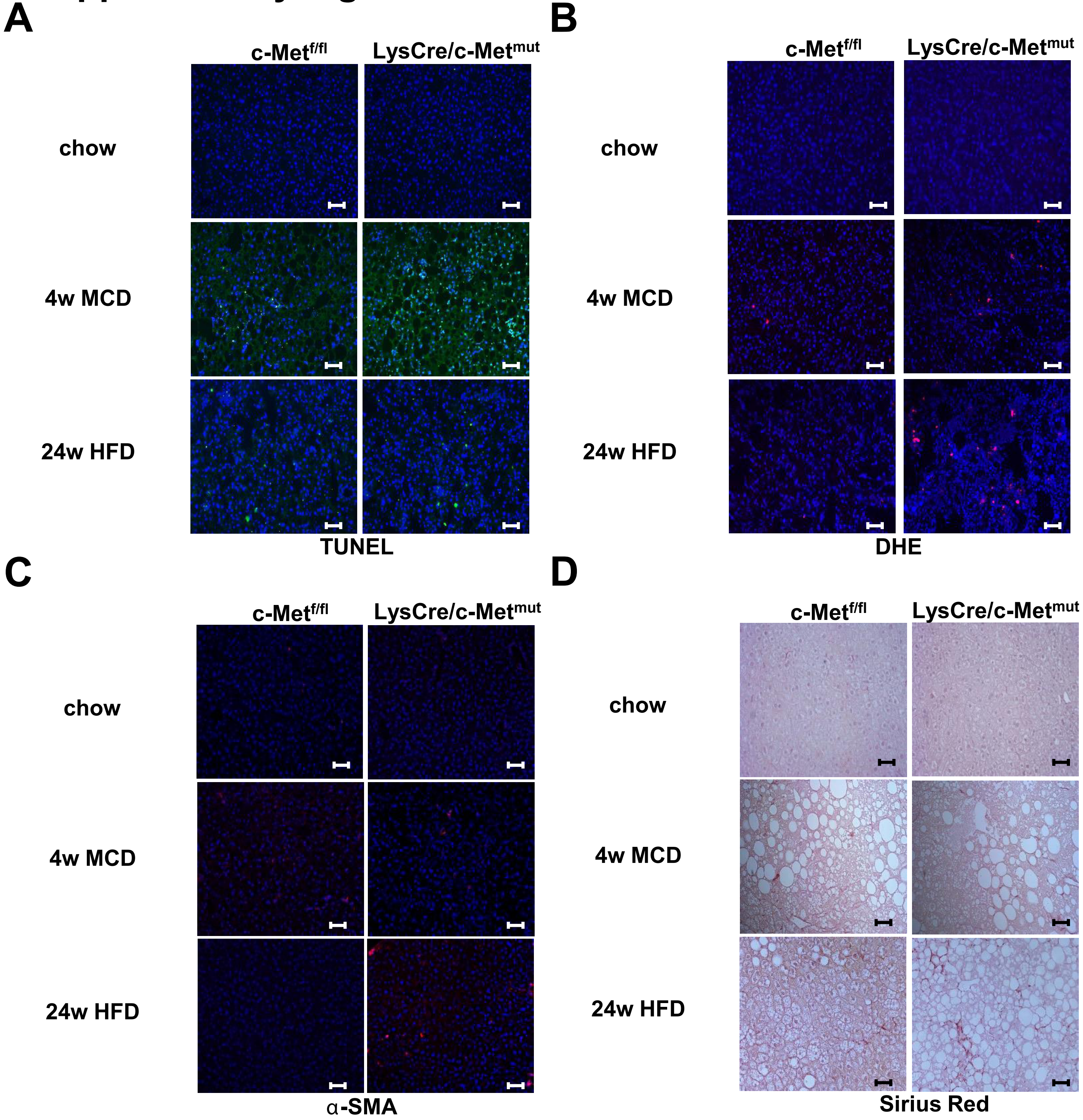

Supplementary Figure 4

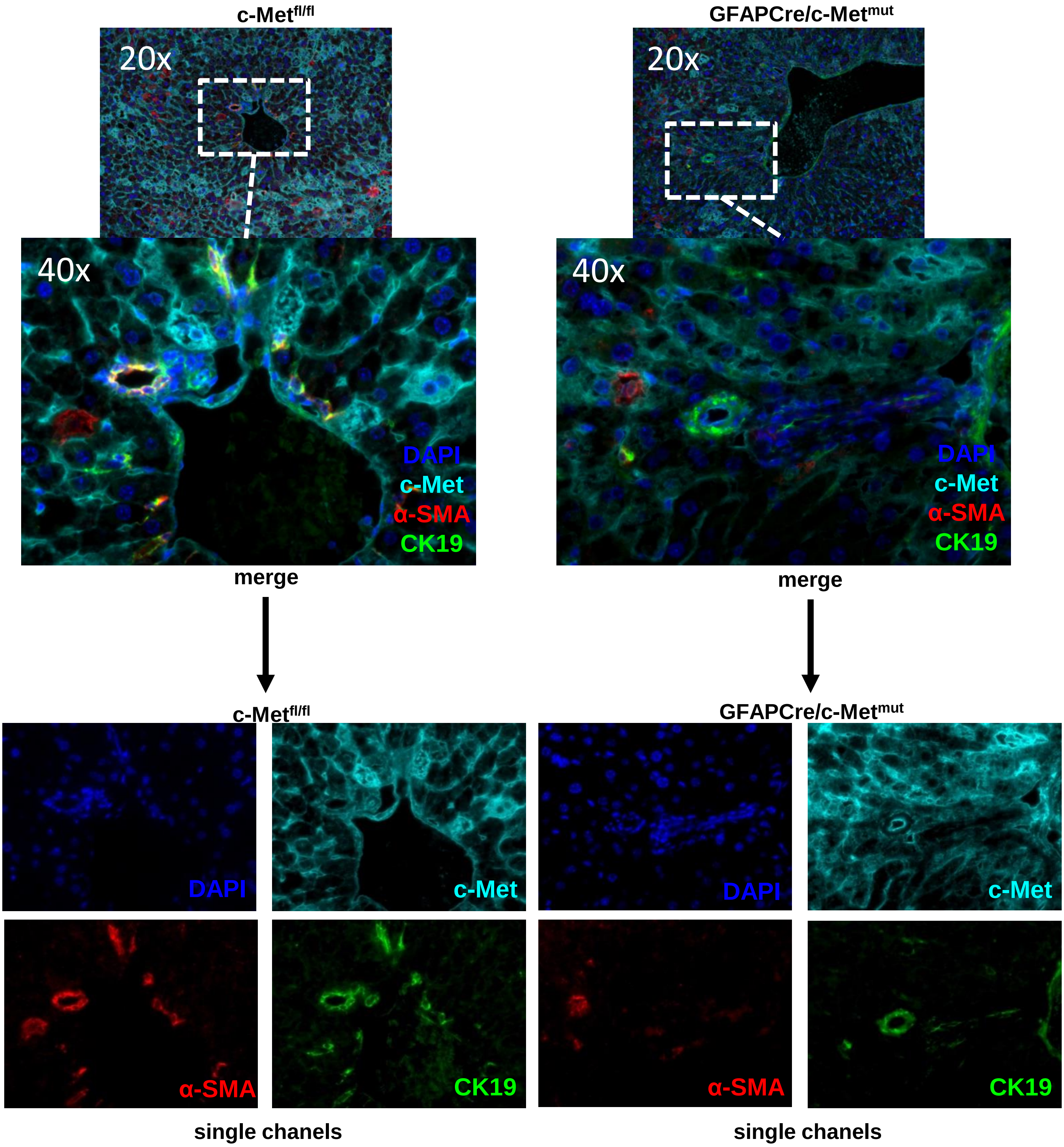

Supplementary Figure 5

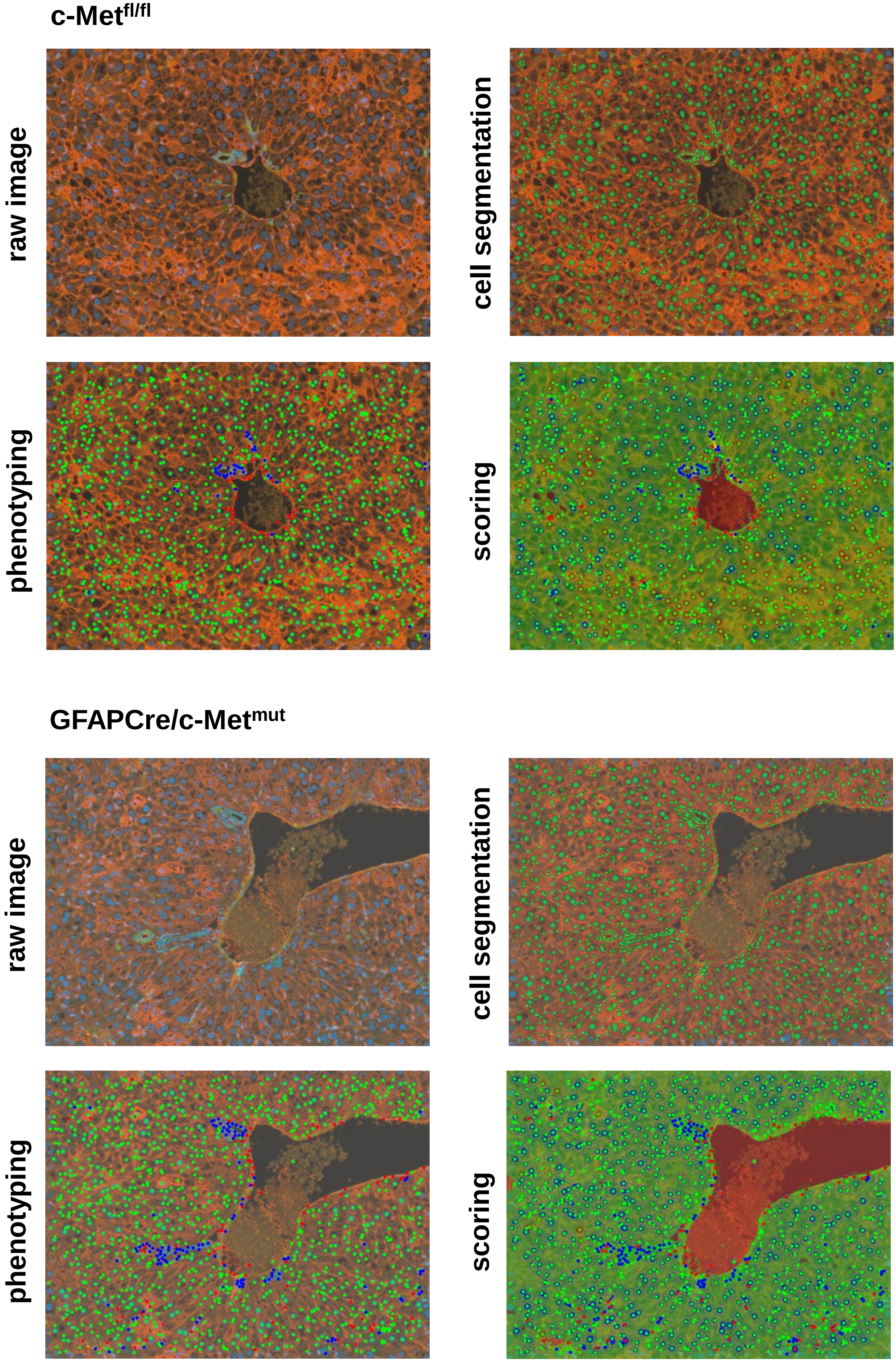

Supplementary Figure 6

A

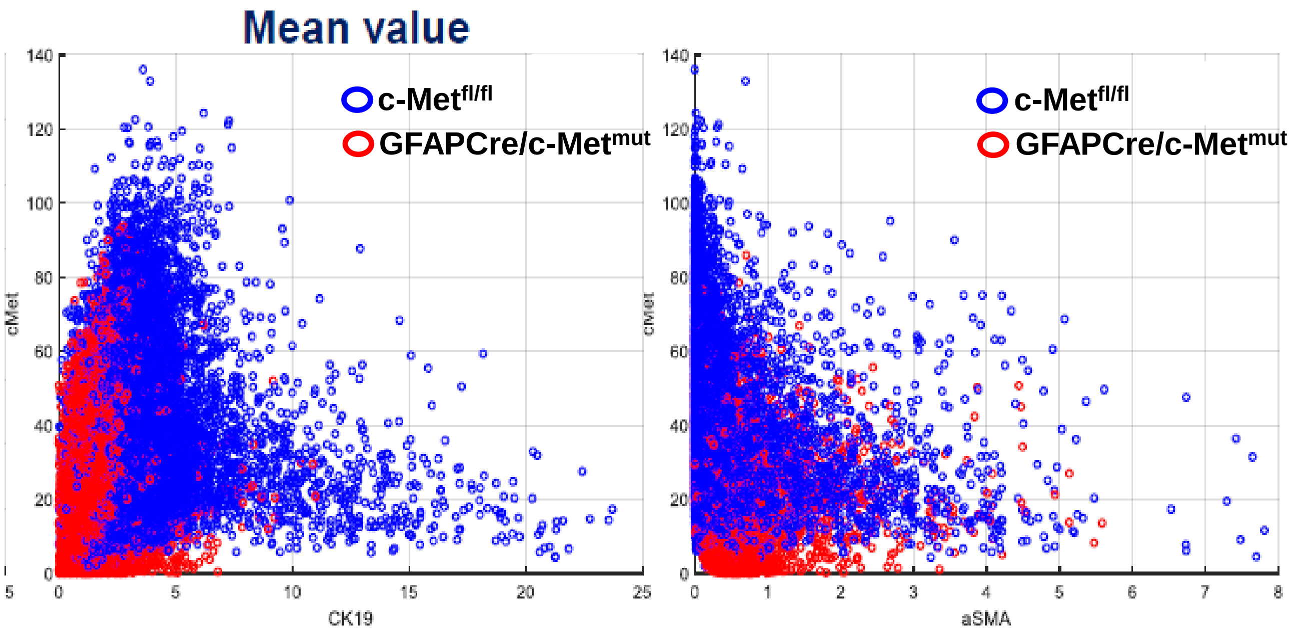

B

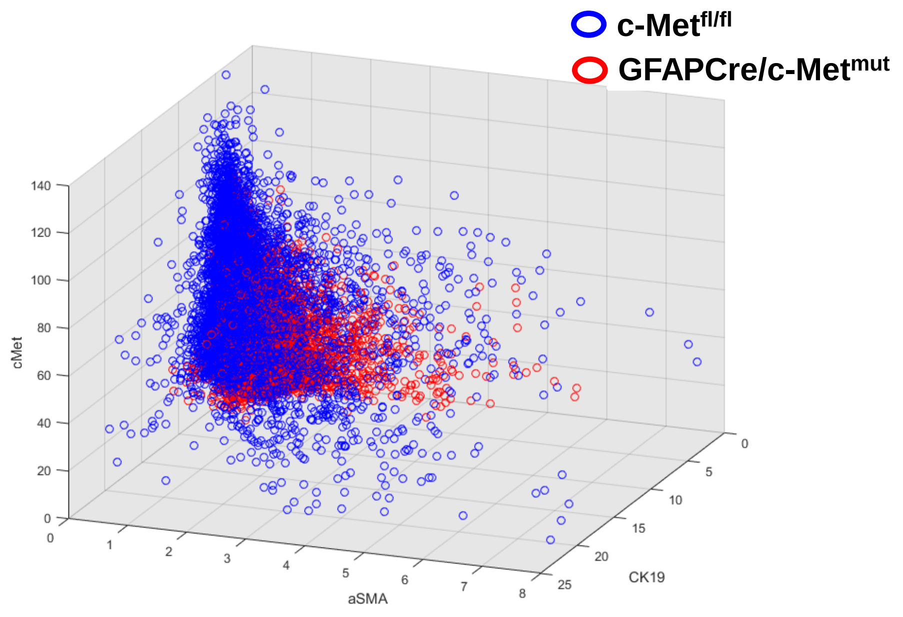

Supplementary Figure 7

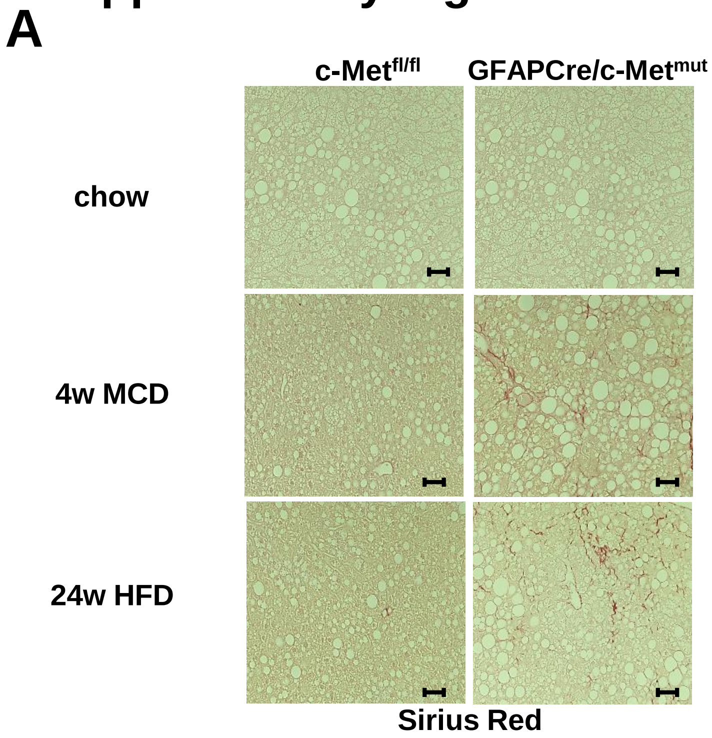

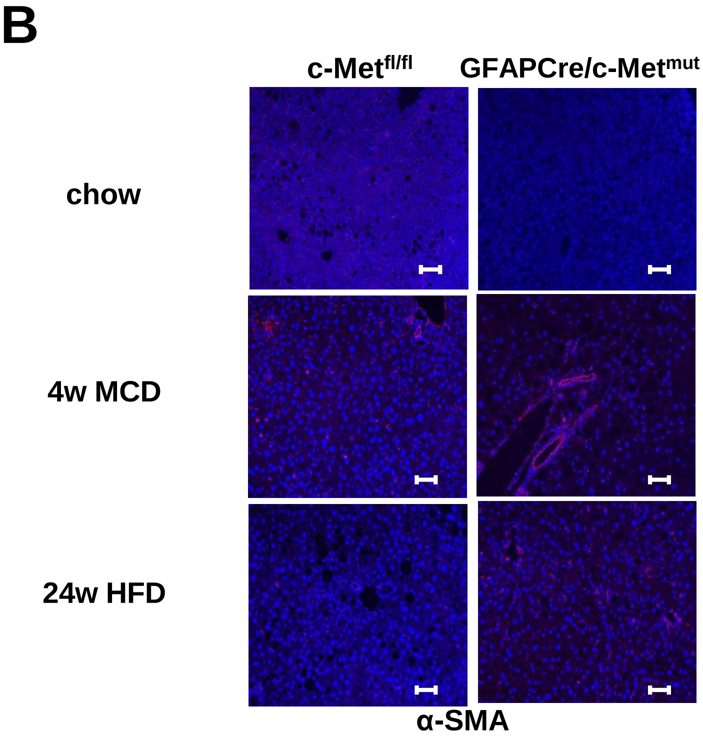

Supplementary Figure 8

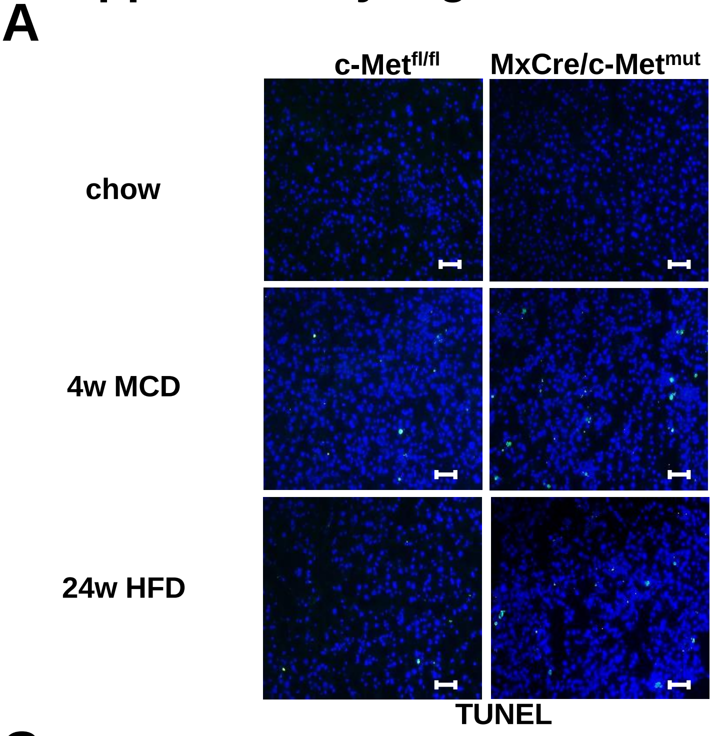

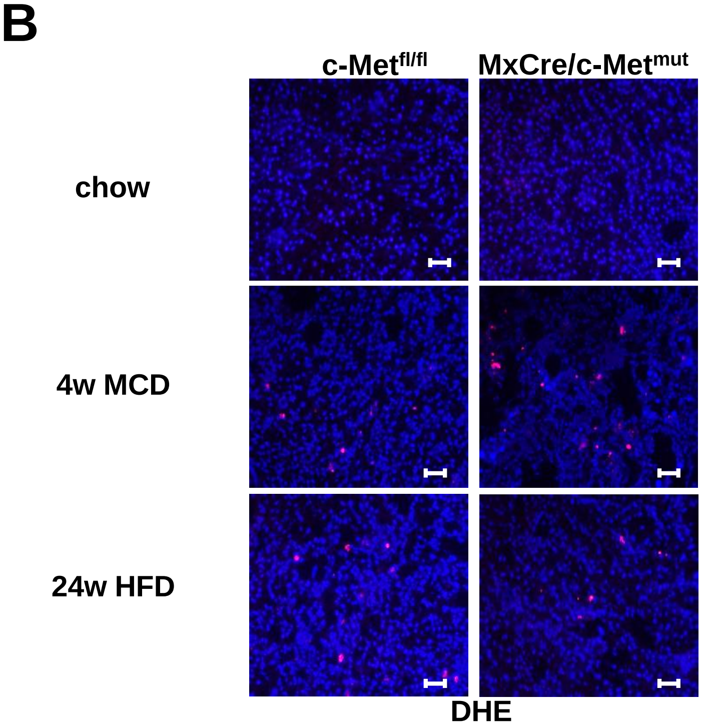

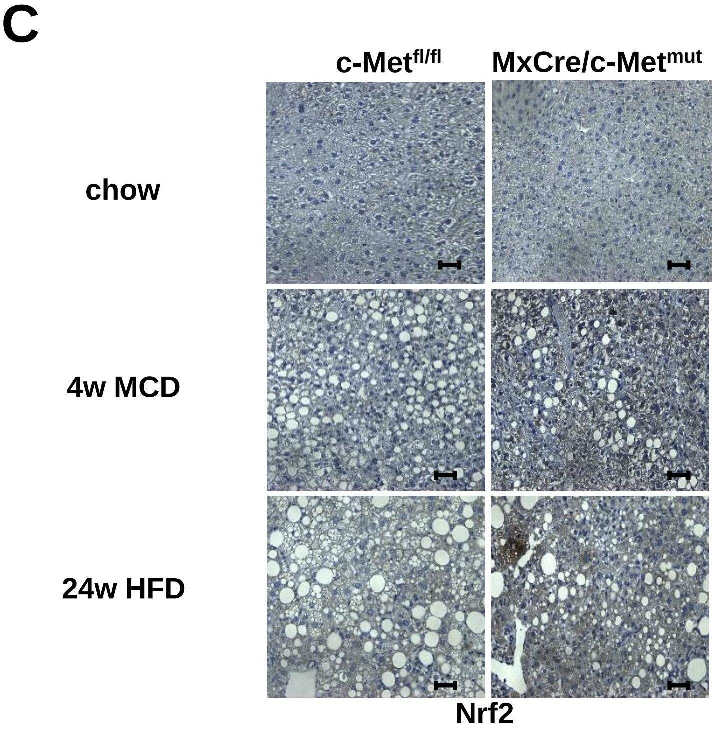

Supplementary Figure 9

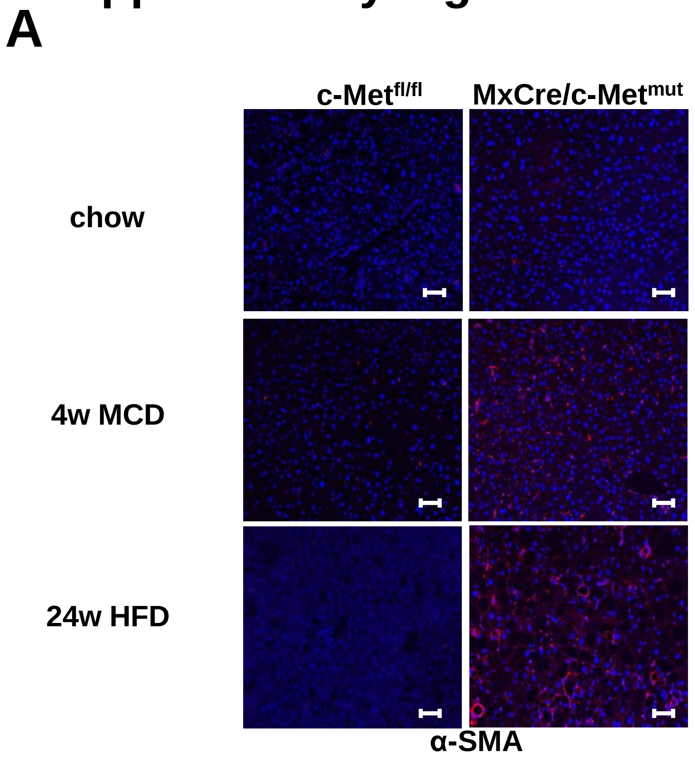

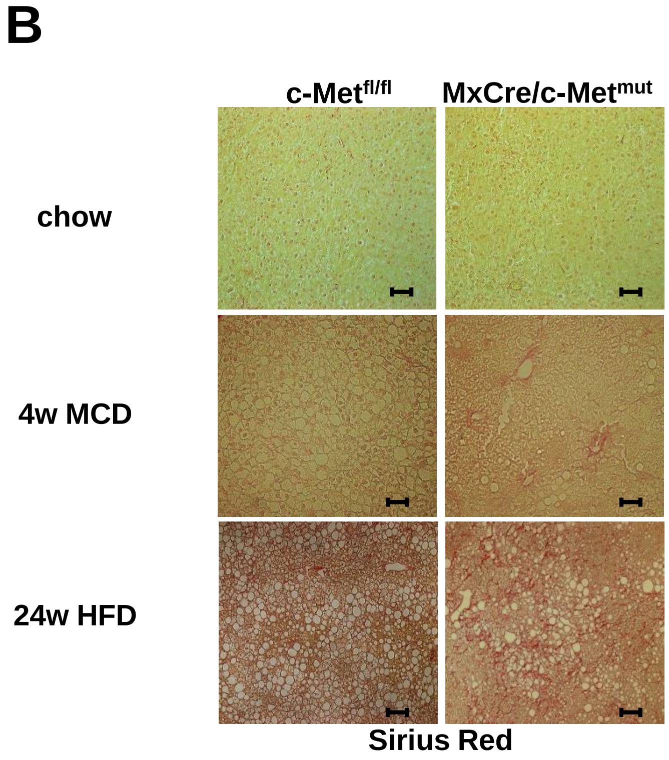

Supplementary Figure Legends

Supplementary Figure 1:

A) Genomic PCR genotyping using oligos for recombined and non-recombined allele. Comparison of knockout PCR products using c-Met$^{fl/fl}$ and LysCre/c-Met^{mut} mice DNA of isolated primary hepatocytes, α-SMA^{+}/CK19^{+} cells and bone marrow-derived immune cells. GFAPCre/c-Met^{mut} mice DNA of isolated primary hepatocytes, Kupffer cells/macrophages and bone marrow-derived immune cells. MxCre/c-Met^{mut} mice DNA of isolated primary hepatocytes, α-SMA^{+}/CK19^{+} cells and Kupffer cells/macrophages. The 500bp product represents the c-Met$^{fl/fl}$ allel, the 800bp product shows the c-Met^{mut} genotype. **B)** Comparison of knockout PCR products using c-Met$^{fl/fl}$ and LysCre/c-Met^{mut} mice DNA of isolated primary Kupffer cells/macrophages. The 500bp product represents the c-Met$^{fl/fl}$ allel, the 800bp product shows the c-Met^{mut} genotype. **C)** Comparison of knockout PCR products using c-Met$^{fl/fl}$ and GFAP/c-Met^{mut} mice DNA of isolated primary α-SMA^{+}/CK19^{+} cells. The 500bp product represents the c-Met$^{fl/fl}$ allel, the 800bp product shows the c-Met^{mut} genotype. **D)** Comparison of knockout PCR products using c-Met$^{fl/fl}$ and Mx/c-Met^{mut} mice DNA of isolated primary bone marrow-derived immune cells. The 500bp product represents the c-Met$^{fl/fl}$ allel, the 800bp product shows the c-Met^{mut} genotype.

Supplementary Figure 2:

Representative gating of the analysis of intrahepatic CD4^{+} and CD8^{+} T cells in c-Met$^{f/fl}$ animals after HFD treatment for 24 weeks. Cells are gated FSC/SSC, FSC-W/FSC-A, live dead/CD45^{+}, CD4^{+} or CD8^{+}.

Supplementary Figure 3:

Representative photographs of **A)** TUNEL, **B)** DHE, **C)** α-SMA and **D)** Sirius Red stained liver sections of c-Met$^{f/fl}$ and LysCre/c-Met^{mut} mice after chow, MCD (4 weeks) or HFD (24 weeks) feeding. (Scale bars 100µm, magnification 200x).

Supplementary Figure 4:

Images of multiplexed immunofluorescence stained liver sections (DAPI, c-Met, alpha-SMA, CK19) of c-Met$^{f/fl}$ and GFAPCre/c-Met^{mut} animals. Magnification 200x and 400x. Together with the corresponding images of the single channels of the

multiplexed immunofluorescence stained liver sections (DAPI, c-Met, alpha-SMA, CK19) of c-Met$^{f/fl}$ and GFAPCre/c-Met^{mut} mice. Magnification 400x.

Supplementary Figure 5:

Scans of the multispectral image acquisition and analysis showing the raw image, cell segmentation, phenotyping and scoring of a c-Met$^{f/fl}$ and GFAPCre/c-Met^{mut} mouse.

Supplementary Figure 6:

A) 2D and **B)** 3D scattered plots based on the analysis of the mean fluorescent intensity of c-Met$^{f/fl}$ and GFAPCre/c-Met^{mut} animals with MATLAB R2016b (MathWorks, Natick, USA).

Supplementary Figure 7:

Representative images of **A)** Sirius Red stained liver sections and **B)** of α-SMA stained liver sections of c-Met$^{f/fl}$ and GFAPCre/c-Met^{mut} mice after dietary treatment (chow, MCD 6 weeks, HFD 24 weeks). Scale bars 100µm, magnification 200x.

Supplementary Figure 8:

Representative photographs of **A)** TUNEL, **B)** DHE and **C)** Nrf2 stained liver sections of c-Met$^{f/fl}$ and MxCre/c-Met^{mut} mice after chow, MCD (4 weeks) or HFD (24 weeks) feeding. (Scale bars 100µm, magnification 200x).

Supplementary Figure 9:

Representative photographs of **A)** α-SMA and **B)** Sirius Red stained liver sections of c-Met$^{f/fl}$ and MxCre/c-Met^{mut} mice after chow, MCD (4 weeks) or HFD (24 weeks) feeding. (Scale bars 100µm, magnification 200x).

c-Met signaling protects from non-alcoholic steatohepatitis (NASH)-induced fibrosis in different liver cell types

Table of contents:

Supplementary Material and Methods: 3

Genomic PCR genotyping 3

Blood collection 3

Serum analysis 3

Glucose tolerance test 3

Hepatic triglycerides 3

Hydroxyprolin assay 3

NAFLD activity score 4

Histology, Oil Red O, Sirius-red 4

TUNEL assay 4

Immunohistochemical Nrf2 and c-Met staining 4

Immunofluorescent stainings (α-SMA, DHE) 5

Multiplexed immunofluorescence 5

Multispectral image acquisition and analysis 6

c-Met knockout PCR 7

Gene expression analysis by Real-Time PCR 7

Isolation of cells and flow cytometry 7

SDS PAGE and western blot 7

Quantification and statistics 8

Supplementary Tables: 9

Supplementary Table 1: Primary antibodies for immunofluorescent stainings and western blot 9

Supplementary Table 2: Oligonucleotides for Real-Time PCR analysis 10

Supplementary Table 3: Antibody Panel used for FACS Analysis 11

Supplementary Table 4: Protein lysisbuffer for SDS PAGE 12

References 13

Supplementary Material and Methods:

Genomic PCR genotyping

Comparison of knockout PCR products using c-Met$^{fl/fl}$ and LysCre/c-Met^{mut} mice DNA of isolated primary Kupffer cells, GFAPCre/c-Met^{mut} mice DNA of isolated α-SMA^{+}/CK19^{+} cells and MxCre/c-Met^{mut} mice DNA of isolated bone-marrow derived immune cells as template. The 500bp product represents the c-Met$^{fl/fl}$ allel, the 800bp product shows the Cre specific c-Met^{mut} genotype.

Blood collection

For retro-orbital blood collection mice were shortly anaesthetised with isoflurane and blood was collected via a glass capillary. Blood samples were centrifuged at 10.000 rpm for 5 min and serum was stored at −80°C.

Serum analysis

For serum glucose, aspartat aminotransferase (AST) and alanine aminotransferase (ALT) levels serum was processed by the Central Laboratory Facility of the University Hospital RWTH Aachen.

Glucose tolerance test

Mice were fasted for 6 hours and blood glucose was measured every 15 minutes for 2 hours following intraperitoneal administration of 2 g/kg glucose.

Hepatic triglycerides

To measure hepatic triglyceride levels 20 mg snap frozen liver tissue was homogenized in 1 ml buffer (10 mM Tris, 2 mM EDTA, 0,25 M sucrose, pH 7,5). A standard curve was calculated in accordance with the manufacturer’s instructions of the Instruchemie liquicolor mono Kit (Instruchemie, Delfzijl, Netherlands). 200 µl kit reagent were added to 2 µg homogenate or to the standard solution and incubated for 45 minutes at room temperature. OD was measured at 492 nm.

Hydroxyprolin assay

For the quantification of liver fibrosis the content of the collagen-specific amino acid hydroxyproline was measured by colorimetric analysis.

NAFLD activity score

A histopathological validation was performed via a NAFLD activity score (NAS) as described by Kleiner *et al.*[1] and Hubscher *et al.*[2].

Histology, Oil Red O, Sirius-red

Livers were fixed in 4% formaldehyde, embedded in paraffin, cut and hematoxylin and eosin stained.

For Sirius-red staining the paraffin sections were cut and incubated in a 0.1% Sirius red and 0.1% picric acid (pH 2.0) solution for 1 h at room temperature.
After that slides were incubated in 0.1 M HCl for 5 minutes, treated with an ascending ethanol series and covered with Roti-Histokit (Roth, Karlsruhe, Germany). 10 Images per slide were analysed under polarized light. Pictures of the Sirius-red positive area were taken in a 400x magnification and analysed via colour error measurement using the open source software Image-J.

Oil Red-O staining was performed with frozen sections. Sections were fixed in formalin, washed in PBS 3x 5 min. and stained in Oil Red-O staining solution (Sigma, Steindorf, Germany). Nuclei were counterstained with haematoxylin.

TUNEL assay

5-µm liver cryosections were fixed with 4% paraformaldehyde and washed with PBS. After 10 min. incubation in 3% H_2O_2 methanol, slides were incubated in sodium citrate (0.1%) for 2 min. and washed again in PBS. Substrate mixture was applied according to the manufacturer's instructions of the TUNEL assay kit (Roche, Mannheim, Germany).

Immunohistochemical Nrf2 and c-Met staining

Paraffin embedded liver sections were cut with a thickness of 5-µm. Sections were deparaffinized and rehydrated. Antigen retrieval was performed by boiling in sodium citrate trisodium salt dehydrate and afterwards incubated for 10 min. in 1,5% hydrogen peroxide. The primary antibody was incubated overnight (Nrf2 mAb, Genetex, GTX103322, c-Met mAb, Santa Cruz, sc-161R) in 1% mouse serum dissolved in PBS (PAA, Vienna, Austria) containing 0,02% sodium acetat (Sigma Aldrich). The second antibody was incubated for 1 h at room temperature. Afterwards slides were incubated with streptavidin ABC-alkaline phosphatase, colour was developed with peroxidase DAB followed by hematoxylin staining.

Immunofluorescent stainings (α-SMA, DHE)

Liver tissue in OCT was cut into 5-µm sections, air-dried and fixed in 4% PFA (paraformaldehyde). Primary antibodies (

Supplementary Table 1: Primary antibodies for immunofluorescent stainings) were incubated for 1h at room temperature in 1% mouse serum in PBS (PAA, Vienna, Austria) containing 0,02% sodium acetat (Sigma Aldrich). Alexa Fluor 594–conjugated or AlexaFluor 488–conjugated secondary antibodies (Molecular Probes, Boston, MA) were used for final detection.

Nuclei counterstaining was performed with DAPI (Vector Laboratories/Axxora, Loerrach). Images at a 400x magnification were taken with the AxioImager Z1 microscope (Carl Zeiss, Jena, Germany). At least 10 photographs per mouse liver with at least 4 animals per group and treatment were included to the analysis.

Multiplexed immunofluorescence

Formalin-fixed paraffin-embedded tissues were cut in 4 µm thick sections and mounted onto Super-Frost Plus slides (Thermo Fisher Scientific, Karlsruhe, Germany). Prior to the multiplex staining the sections were pre-treated with green and red light (515 nm and 630 nm) overnight in the FluorVivo™ 300 (INDEC BioSystems, Los Altos, USA) to reduce autofluorescence without affecting the tissue integrity. Slides were heated at least for 1 h in a dry oven at 60°C, immersed in 100% xylene to deparaffinize and rehydrated with a series of graded ethanol into distilled water. The tissue was fixed in 10% neutral-buffered formalin for 20 min, after which the antigen retrieval was performed using the AR6 buffer (PerkinElmer, Hopkinton, USA) for 20 min in a 98°C water bath. Blocking was performed for 30 min with 10% donkey serum (Abcam plc, Cambridge, UK). The first primary antibody for CK19 (Santa Cruz sc-33111, dilution 1:800) was incubated for 1 h in a humidified chamber at room temperature followed by detection using anti-goat antibody (abcam ab97110, dilution 1:300). Visualization of CK19 was accomplished using the TSA Plus Cyanine 5 Kit (PerkinElmer, Hopkinton, USA, dilution 1:50), after which the antigen retrieval step was repeated. In a serial fashion, the slides were then incubated with the primary antibody for c-Met (Santa Cruz sc-161, dilution 1:100) for 1 h in a humidified chamber at room temperature, followed by detection using anti-mouse antibody (Santa Cruz sc-2005, dilution 1:300), after which c-Met was visualized

using TSA Plus Cyanine 3 Kit (PerkinElmer, Hopkinton, USA, dilution 1:50). The slides were again placed in AR6 buffer and subject to heat induced antibody complex removal. Afterwards the sections were incubated with the last primary antibody directed against α-SMA (abcam ab32575, dilution 1:100), for 1 h in a humidified chamber at room temperature, followed by detection using the anti-rabbit antibody (Cell Signaling #7074S, dilution 1:300). Visualization of α-SMA was accomplished using the TSA Plus Fluorescein Kit (PerkinElmer, Hopkinton, USA, dilution 1:50). Counterstaining of cell nuclei was performed using DAPI (Thermo Fisher Scientific, Karlsruhe, Germany) and the sections were embedded with Mowiol 4-88 (Roth, Karlsruhe, Germany).

Multispectral image acquisition and analysis

Whole multiplex stained sections were scanned automatically using the Vectra 3.0 Automated Quantitative Pathology Imaging System (PerkinElmer, Hopkinton, USA). Whole slide scans were acquired with the 10x objective and multispectral images with the 20x objective. In order to establish a spectral library that enables the spectral information to be reliably unmixed and quantified, the system requires individual examples of each fluorophore emission spectra on the respective specific tissue type as well as a representative autofluorescence spectrum of an unstained sample. The Vectra captures fluorescent spectra with 20 nm wavelength intervals in a range from 420 nm to 720 nm and combines these captures into a single image stack, while retaining the spectral signature of the used fluorophores. Image files created by the Vectra were analyzed using the InForm 2.3 analysis software (PerkinElmer, Hopkinton, USA). InForm employs a pattern recognition learning algorithm that supports a tissue and cell classifier tool, which was trained to segment and phenotype individual cells and generate a pixel based intensity report for all markers. Supplementary Figure 5 shows the training process applied to the system for individual analysis. The upper left panel first shows the raw data image. After that the computer performed a cell segmentation approved by the performing scientist. By giving the computer few examples what segmented cell belongs to which cell type, the computer implements an automated phenotyping for the whole image, again controlled by the scientist. The last step is the computational calculation which cell type expresses the stained markers in a specific calculated intensity. Based on the mean fluorescent intensity a positivity threshold for each cell type was determined for the scoring of each

marker, which is depicted in 2D and 3D scattered plots using MATLAB R2016b (MathWorks, Natick, USA).

c-Met knockout PCR

c-Met deletion was tested by reverse transcription PCR. RNA from isolated Kupffer cells (c-Met$^{fl/fl}$, LysCre/c-Met^{mut}) or bone-marrow cells (c-Met$^{fl/lf}$, MxCre/c-Met^{mut}) were analysed using primers that detect a c-Met deletion on exon 15 in the c-Met gene. The band intensity was evaluated densitometrically.

Gene expression analysis by Real-Time PCR

mRNA of whole liver extracts was isolated from cryopreserved tissue with the peqGOLD RNAPure kit (Peqlab, Erlangen, Germany) according to the manufacturer's instructions. After DNAse digestion with the DNAse I kit (Invitrogen, Karlsruhe, Germany) 500-1000 ng of total mRNA were used for cDNA synthesis by reverse-transcription with the Omniscript reverse-transcription kit (Qiagen, Hilden, Germany) according to the manufacturer's instructions. cDNA was used in Real-Time PCR (Applied Biosystems, Foster City, CA). Detection of the expression of different genes of interest was performed using SybrGreen tqPCR Supermix (Invitrogen) (Primer sequences of genese of interest can be found in Supplementary Table 2. At least 6 animals per group were analyzed.

Isolation of cells and flow cytometry

For the analysis of intrahepatic leukocytes, whole livers were perfused with 10 ml phosphate-buffered saline (PBS), cut into pieces and digested with collagenase IV (Worthington) at 37°C for 30 minutes. Liver extracts were then filtered through a 70 µm cell strainer and cells were stained with fluorochrome-labeled monoclonal antibodies (used panel are depicted in Supplementary Table 3: Antibody Panel used for FACS Analysis) for 20 minutes at 4°C. Flow cytometric analysis were then performed via the BD Fortessa (BD Biosciences, Heidelberg, Germany). Data were analyzed with the FlowJo software (TreeStar, Ashland, USA).

SDS PAGE and western blot

Snap frozen liver tissue was liquidated with lysis buffer (Supplementary Table 4: Protein lysisbuffer for SDS PAGE). The obtained protein lysate was heat denaturated at 95°C for 5 min. in double-strength sodium dodecyl sulphate sample buffer containing dithiothreitol before resolution in 10% SDS-PAGE. Prior to the

primary antibody incubation, membranes were incubated with anti-α-SMA (ab32575, Abcam, Cambridge, UK), and anti-GAPDH (MCA4739, AbD serotec, Hercules, CA) antibodies. Secondary antibodies were HRP-linked anti-rabbit immunoglobulin G (7074S, Cell signaling, Frankfurt, Germany) and HRP-linked anti-rat (559286, BD Pharmingen, Heidelberg, Germany). Visualization of the antigen-antibody complex was achieved by the use of the ECL Chemiluminescence Kit (GE Healthcare, Buckinghamshire, UK).

Quantification and statistics

Results were expressed as mean±SEM. Significant p values were measured by the Student's t test. A value of $p<0.05$ was considered significant (* $p<0.05$, ** $p<0.01$, *** $p<0.001$).

Supplementary Tables:

Supplementary Table 1: Primary antibodies for immunofluorescent stainings and western blot

immunoreactivity	dilution	species	manufacturer
α-SMA	1:100	Rabbit	Abcam, Cambridge, UK, Cat. No ab32575
Dihydroethidium(hydroethidine) (DHE)	1:500	-	Invitrogen, Carlsbad, CA, Cat. No D23107
GAPDH	1:20.000	Mouse	AbD serotec, Oxford, UK, Cat. No. MCA4739

Supplementary Table 2: Oligonucleotides for Real-Time PCR analysis

oligonucleotide	sequence	manufacturer
**GAPDH**	*forward:* TGT TGA AGT CAC AGG AGA CAA CCT<br>*reverse:* AAC CTG CCA AGT ATG ATG ACA TCA	MWG, Ebersberg, Deutschland
**TGF-β**	*forward:* CAG CTT TGC TCA CTG CAG TAC A<br>*reverse:* CTT GGC CGC CTC TAA CGA TA	MWG, Ebersberg, Deutschland
**TNF-α**	*forward:* AGC ACA GAA AGC ATG ATC CG<br>*reverse:* CCC GAA GTT CAG TAG ACA GAA GAG	MWG, Ebersberg, Deutschland
**IL-6**	*forward:* CTG CAA GAG ACT TCC ATC CAG<br>*reverse:* AGT GGT ATA GAC AGG TCT GTT GG	MWG, Ebersberg, Deutschland
**α-SMA**	*forward:* ATG AAG CCC AGA GCA AGA GA<br>*reverse*: ATG TCG TCC AGT TGG TGA T	MWG, Ebersberg, Deutschland
**Collagen1α**	*forward:* GCT ACT ACC GGG CCG ATG ATG C<br>*reverse:* CCT TCG GGG CTG CGG ATG TTC	MWG, Ebersberg, Deutschland

Supplementary Table 3: Antibody Panel used for FACS Analysis

panel	Antibody (anti-mouse)	conjugate	manufacturer
**mouse**	CD45	APC-Cy7	BD Pharmingen, Heidelberg, Germany, Cat. No 557659
	CD4	FITC	eBioscience, Santa Clara, CA, Cat. No 11-0042-82
	CD8	PerCP-Cy5.5	BD Pharmingen, Heidelberg, Germany, Cat. No 551162
	7AAD dye		Sigma Aldrich, St Louis, MO
**human**	c-Met	FITC	eBioscience, Santa Clara, CA, Cat. No 11-8858-42
	CD16	APC-H7	eBioscience, Santa Clara, CA, Cat. No 17-0168-42
	CD56	APC	eBioscience, Santa Clara, CA, Cat. No 17-0566-42
	CD66b	APC	eBioscience, Santa Clara, CA, Cat. No 17-0666-42
	CD19	APC	eBioscience, Santa Clara, CA, Cat. No 17-0198-42

Supplementary Table 4: Protein lysisbuffer for SDS PAGE

1 M	NaCl
0,01 M	EGTA
0,5 M	EDTA (pH 8)
1 M	NaH_2PO_4
1 M	Tris (pH 7,5)
1 M	NaF
10x	Triton
0,1 M in EtOH	PMSF
1 mM	Na_3VO_4
In H_2O dest.	

References

[1] Kleiner DE, Brunt EM, Van Natta M, Behling C, Contos MJ, Cummings OW, et al. Design and validation of a histological scoring system for nonalcoholic fatty liver disease. Hepatology 2005;41:1313-1321.
[2] Hubscher SG. Histological assessment of non-alcoholic fatty liver disease. Histopathology 2006;49:450-465.
